# Mechanisms of Arsenic Interaction in *Bacillus subtilis* and Related Species with Biotechnological Potential

**DOI:** 10.3390/ijms262110277

**Published:** 2025-10-22

**Authors:** Luz I. Valenzuela-García, María Teresa Alarcón-Herrera, Elizabeth Cisneros-Lozano, Mario Pedraza-Reyes, Víctor M. Ayala-García

**Affiliations:** 1 Department of Sustainable Engineering, Advanced Materials Research Center (CIMAV), Durango P.C. 34147, Mexico; luz.valenzuela@cimav.edu.mx; 2 Faculty of Chemical Sciences, Juárez University of Durango State, Durango P.C. 34120, Mexico; 66554@alumnos.ujed.mx; 3 Department of Biology, University of Guanajuato, Guanajuato P.C. 36050, Mexico; pedrama@ugto.mx

**Keywords:** *Bacillus*, environmental isolates, arsenic detoxification, molecular mechanisms, resistance, biotechnology, bioremediation, genetic regulation

## Abstract

Arsenic (As) toxicity drives the evolution of resistance mechanisms in environmental microorganisms. Bacteria of the *Bacillus* genus are frequently identified in isolates from arsenic-contaminated sites, highlighting the importance of understanding the molecular mechanisms related to this bacterial genus. *Bacillus subtilis*, a soil microorganism and Gram-positive model paradigm, employs multiple strategies to counteract As toxicity, including biosorption, redox transformation, active efflux, and inducible genetic regulation. This review provides a comprehensive analysis of the physiological and molecular mechanisms involved in arsenic response in *B. subtilis* and related species, focusing on the *ars* and *ase* operons. The *ars* operon, located within the mobile SKIN element, encodes a reductase (ArsC), an Acr3-type efflux pump (ArsB), a carbon–arsenic lyase (ArsI/YqcK), and a transcriptional repressor (ArsR), all co-regulated in response to arsenic. In turn, the *ase* operon contributes to resistance via an ArsB-type efflux system (AseA) and its own regulatory protein (AseR) but lacks an arsenate reductase. Additionally, genes such as *aioAB*, *arrAB*, and *arsD* are discussed, along with evidence for extracellular detoxification and cell surface immobilization of As. Studies on environmental *Bacillus* species are examined, pointing out the evolutionary implications of As resistance and the biotechnological potential for remediation of contaminated sites.

## 1. Introduction

*Bacillus subtilis* is a Gram-positive model organism that has gained significant interest in biotechnological applications due to its robust physiological traits, including biofilm formation, spore production and its GRAS (Generally Recognized As Safe) designation that belongs to the Firmicutes phylum [1]. Notably, ~1000 of its 4100 genes (approximately 25%) are orthologues to those from the most extensively studied Gram-negative bacterium *Escherichia coli*; however, it has been estimated that there are about one billion years of evolutionary divergence between them, leading to notable differences in environmental adaptation [2]. Genes involved in essential functions for bacterial life, such as transcription and translation, metabolic functions, murein biosynthesis and cell division, general stress proteins, and membrane transport are shared by both prokaryotes [2]. Non-ortholog genes in *B. subtilis* include prophage-related genes and genes for secondary metabolism and antibiotic production, sporulation and competence, transport proteins, and interaction and adaptation to plant environments [3,4,5]. These genetic differences confer *B. subtilis’* adaptive characteristics to diverse environments that are of biotechnological interest [2].

The *Bacillus* genus includes species involved in carbon and nitrogen cycling, human and livestock pathogens such as *B. anthracis* and *B. cereus*, and insect pathogens as *B. thuringiensis*; however, most species within this genus are non-pathogenic [6]. *B. subtilis* encompasses a set of subspecies and closely related species, collectively referred to as the *Bacillus subtilis* species complex [6]. Members of this complex share 99% or higher 16S gene sequence identity, making them difficult to distinguish phenotypically based on morphology, physiology, or biochemical characteristics, as these traits may vary significantly among strains. For instance, some *B. subtilis* strains can grow under anaerobic conditions or at temperatures above 56 °C, while others cannot, potentially leading to confusion with other species within the complex [6]. Species within this complex include *Bacillus amyloliquefaciens*, *Bacillus atrophaeus*, *Bacillus axarquiensis*, *Bacillus mojavensis*, *Bacillus inaquosorum*, *Bacillus licheniformis*, *Bacillus malacitensis*, *Bacillus sonorensis*, *Bacillus tequilensis*, *Bacillus vallismortis,* and *Bacillus velezensis* [6].

Among the *B. subtilis* strains widely used in research are those carrying auxotrophies for threonine (strain 23), nicotinic acid (strain 122), and tryptophan (strains 166, 167, and 168), generated via X-ray mutagenesis. However, the parental wild-type (WT) strain from which they were derived was lost, although all these mutants share the same genetic origin. Of these, strain 168 is the most used as a WT standard reference strain across multiple research groups and is classified as *B. subtilis* subsp. *subtilis,* whereas strain W23, which differs both genotypically and phenotypically from strain 168, is referred to as *B. subtilis* subsp. *Spizizenii* [7]. Genome sequencing of *B. subtilis* strain 168 revealed the presence of cryptic prophage elements such as PBSX, SPβ, and phage-like elements such as *SKIN*, integrated at different chromosomal loci. Therefore, it is feasible that horizontal gene transfer via bacteriophage infection has contributed to the acquisition of specific genes in this strain [2].

Throughout its evolutionary trajectory, *Bacillus* has developed genetic mechanisms to adapt to various environmental stressors, including the ability to utilize diverse carbon sources, secrete extracellular enzymes, synthesize antibiotics, and thrive in potentially toxic environments, particularly those contaminated with metals and metalloids. Arsenic is a toxic metalloid which has been found to be part of abundant crustal minerals existing for over 3 billion years [8]. To counteract As toxicity, microorganisms have developed efficient detoxification and tolerance mechanisms. This review focuses on the mechanisms employed by bacteria of the *Bacillus* genus to withstand and proliferate in environments polluted by arsenic, highlighting the main differences with other bacteria less distributed in natural environments such as *E. coli*.

## 2. Arsenic Toxicity and Cellular Interactions

### 2.1. Chemical Forms and Public Health Impact

Arsenic (As) is one of the most naturally abundant elements in the Earth’s crust and oceans, particularly in areas of high hydrothermal activity, mineral deposits, Cenozoic sediments, and carbon sources along the Pacific Ring of Fire [9]. Its ubiquity in soil environments explains the evolutionary selection for microbial genetic elements capable of mitigating its toxic effects, as elevated arsenic levels are detrimental to cellular function [10].

As belongs to Group VA of the periodic table and possesses five valence electrons. Its most common oxidation states are +3 and +5. It can form inorganic compounds with elements such as oxygen, sulfur, iron, and chlorine, as well as organic compounds containing carbon and hydrogen. Both inorganic and organic forms of As can exist predominantly in the trivalent [As(III)] and pentavalent [As(V)] oxidation states, with the trivalent forms generally exhibiting significantly higher toxicity [11,12,13].

### 2.2. Mechanisms of Toxicity and Detoxification of Arsenic

As toxicity mechanisms vary according to its oxidation state and the biological system in question. As(V), being a molecular analog of phosphate, inhibits oxidative phosphorylation by disrupting energy metabolism. As(III), on the other hand, binds to sulfhydryl groups, impairing protein function and cellular respiration by targeting thiol groups in metabolic enzymes such as pyruvate dehydrogenase and 2-oxoglutarate dehydrogenase. Additionally, As (III) can induce the production of reactive oxygen species (ROS) and cause DNA damage and mutagenesis in mammalian cells [14,15].

Although arsenic is an element that naturally occurs in the Earth’s crust, human activities have led to the release of increasing concentrations of this metalloid to toxic levels in regions and ecosystems where it is not naturally found. The mobility of arsenic, its ability to translocate in plants and bioaccumulate in the food chain, has led to the presence of arsenic in human drinking water and food, increasing exposure doses in humans [16]. Arsenic exposure in humans is associated with health problems such as cancer and cardiovascular, neurological, cardiac, and dermatological disorders [17]. Arsenic detoxification in humans requires enzymatic transformation into the methylated and oxidized forms, monomethyl arsenate [MAs(V)] and dimethyl arsenate [DMAs(V)], which are excreted in the urine. Dimethyl arsenite [DMA(III)] is the precursor of more complex and less toxic organic compounds such as arsenosugars, arsenolipids, and arsenobetaine, which are abundant in aquatic environments but can also be degraded to simpler organic species or inorganic arsenic by microbial action.

Microorganisms have evolved various strategies to cope with As toxicity, including surface adsorption, redox transformation (reduction/oxidation), active efflux, volatilization, and DNA repair mechanisms [18,19,20,21,22]. Certain microorganisms can convert arsenic into various organic and inorganic forms, such as monomethylarsonic acid (MMA), dimethylarsinic acid (DMAA), trimethylarsine (TMA), trimethylarsine fatty alcohols (TMAsFOH), arsenic-containing hydrocarbons (AsHCs), arsenolipids, arsenosugars, and arsenobetaine (AB). Moreover, some microbes can degrade or transform naturally occurring or synthetic arsenical compounds such as phenylarsenicals and roxarsone, leading to the release of inorganic arsenic. Enzymes involved in these transformations include arsenic methyltransferases (ArsM), arsenate reductases (ArsC, ArrAB), arsenite oxidases (AioAB, ArxA), acetyltransferases (ArsN), and arsenic lyases (ArsI), among others [19,23].

While *E. coli*, the Gram-negative bacterial model to study As interactions, is an enterobacteria found in the intestines of animals, the Gram-positive model *B. subtilis* is found in soil, water, and plants [3,24]. Consequently, the molecular mechanisms underpinning As resistance may significantly differ between the two genera. Therefore, the following sections are aimed at describing mechanistic aspects regarding the processing of As conducted on *B. subtilis* and other Firmicutes.

### 2.3. The First Line of Defense: Extracellular Detoxification

The initial factor contributing to As cytotoxicity in *Bacillus* lies in its ability to penetrate the cell barriers, composed of both a lipid membrane and the bacterial cell wall. Thus, As can be immobilized at the cell surface by interacting biomolecules, or alternatively, transformed into a less toxic state by extracellular or periplasmic enzymes, thereby preventing its entry into the cytoplasm [25].

Arsenate [As(V)] is ionized at comparable *pKa* values: 2.2, 7.0, and 11.5 for arsenate and 2.1, 7.2, and 12.7 for phosphate [26]. Due to the molecular similarity between the phosphate group and arsenate, several studies suggest that arsenate may use phosphate transport systems as a route of cellular entry [19,27]. In contrast, arsenite [As(III)] tends to form compounds structurally analogous to hexoses, leading to the hypothesis that its uptake may occur via hexose permeases [21,27,28].

The entry of arsenic into microbial cells is a decisive factor in arsenic toxicity. Upon incorporation into the cell cytoplasm, arsenic can cause DNA damage, interact with the sulfhydryl (-SH) groups of proteins, inhibit the function of enzymes that depend on thiol groups, affect protein folding, and interfere with RNA and protein synthesis [29,30]. Therefore, preventing arsenic from entering cells is the first line of defense in bacteria.

#### 2.3.1. Cell Surface Immobilization

The immobilization of As at the bacterial cell surface involves several processes, including biosorption, surfactant-mediated interaction, and precipitation, all of which serve to prevent arsenic from entering the cell. These mechanisms have been extensively studied due to their potential applications in the bioremediation of As and other heavy metals [31].

Biosorption is a physicochemical phenomenon involving passive interactions with biological materials to bind and concentrate contaminants [32]. During As biosorption, ions interact with cell wall or membrane components such as polysaccharides, proteins, and lipids that possess negatively charged functional groups. This is a passive process, not requiring energy or active cellular metabolism, and can even be carried out by non-viable (dead) cells. The critical factor is the presence of functional groups such as carboxyl, hydroxyl, amino, and phosphonate moieties [33]. Biosorption encompasses various interactions at the cell surface, including ion exchange, electrostatic or covalent complex formation with extracellular polymeric substances (EPSs), and physical adsorption onto the cell envelope.

The capacities of As biosorption have been evaluated in several species of the genus *Bacillus.* At pH 8, *B. subtilis* exhibited a biosorption capacity of 5.14 mg/g of biomass for As(III) and 2.35 mg/g of biomass for As(V) [34]. In contrast, *B. cereus* exhibited a maximum biosorption capacity for As(III) of 32.42 mg/g under optimal conditions, through a spontaneous, endothermic process that followed a Langmuir isotherm model [35]. Another strain of *B. cereus* isolated from a gold mine showed an even higher biosorption capacity of 153.41 mg/g, with hydroxyl, amide, and amine groups identified as the key functional moieties responsible for As binding [36]. Arsenic biosorption has also been found in the biomass of other *B. cereus* strains, including SZ2, W2, and MCC3402 [33,37,38]. Additional strains of the genus *Bacillus* have been tested for As biosorption, including *Bacillus* megaterium strains H3 and UM-123 [39,40], *Bacillus licheniformis* strains B3-15, DAS1, and NDSA24R [40,41,42,43], *Bacillus thuringiensis* strains WS3, A01 [38,44], *B. amyloliquefaciens* NBRISN13 [45], *Bacillus salmalaya* 139SI [46], *Bacillus pacificus* AKS1a [47], and *Bacillus pumilus* [40]. Overall, the biosorption capacities of these strains range from 0.005 mg/g per biomass in *B. pumilus* [40] to 153.4 mg/g of biomass in *B. cereus* SZ2 [36]. Furthermore, surface modification of *B. subtilis* cells by incubation with ferric iron (Fe[III]), which led to a deposition of ferric hydroxide onto the membrane, have shown to enhance As biosorption capacity by up to 9.51 mg/g for As(III) and 12.89 mg/g for As(V) at pH 8 [34]. Another *Bacillus* species, *B. arsenicus* MTCC4380, when immobilized on granular-activated carbon or MnFe_2_O_4_, was able to remove both As(III) and As(V) from wastewater through a spontaneous and exothermic process [48].

In species of the genus *Bacillus*, it has been reported that the biosorption capacity of arsenic is due to the presence of carboxylate, carboxyl, carbonyl, hydroxyl, amide, and amino functional groups on the surface of the biomass, as well as some glycosylated and/or phosphorylated proteins [21,35,36,47]. In addition, the multiple layers of the spores and their specific compositions are very important in the biosorption capacity of metals and metalloids [36]. A kinetic study using *B. subtilis* and *Proteus mirabilis* revealed that the rate-limiting step in As biosorption from contaminated soils is intraparticle diffusion [49].

Another strategy employed by microorganisms to cope with As toxicity is bioprecipitation. This mechanism reduces the concentration of soluble As in the surrounding environment by transforming it into insoluble species, thereby decreasing its ability to permeate biological membranes and its associated toxicity. Certain sulfate-reducing bacteria can precipitate As, generating arsenic sulfide under acidic conditions [50]. This phenomenon was also observed in bacteria isolated from an As-contaminated site, where a microbial consortium predominantly composed of species from the *Caloramator*, *Clostridium*, and *Bacillus* genera was able to remove 65% of soluble As within 150 days of incubation, forming crystalline arsenic sulfide (AsS) [51]. Extracellular polymeric substances (EPSs), such as some saccharides, can also act as reducing agents, and some quinones can act as mediators of electron transfer in the reduction of As(V) to As(III) [52].

#### 2.3.2. Transformation of As by Extracellular Enzymes

The chemical transformation of As by enzymes located in the external surface of the cell membrane also constitutes a mechanism that prevents As from entering the cell. For instance, an arsenite oxidase located in the outer surface of the cell membrane of *Alcaligenes faecalis* has been shown to catalyze the oxidation of arsenite using cytochrome C as an electron acceptor in the presence of azurin [53]. Additional arsenite oxidases have been identified in some β-Proteobacteria isolated from As-rich environments, playing important roles in the respiratory chain, which allows these organisms to utilize arsenic as an energy source [54].

Some species of the *Bacillus* genus have been reported to use As compounds as terminal electron acceptors during anaerobic respiration. Notably, *B. arsenicoselenatis*, an isolate from the arsenic-rich Mono Lake, can reduce arsenate to arsenite under anaerobic conditions, using arsenate as a terminal electron acceptor [55]. Similarly, *Bacillus* sp. strain IIIJ3-1, isolated from As-contaminated water, displays the ability to reduce As(V) to As(III) under anaerobic conditions, putatively employing the arsenate reductase gene *arrA*, which will be discussed in subsequent sections [56]. Other species such as *B. licheniformis* A6 and *B. flexus* As-12 can transform arsenic by oxidizing As(III) to As(V), and the last can also reduce As(V) to As(III) through a cell-associated enzyme activity, although it is unknown whether this activity is carried out by intracellular enzymes or enzymes anchored to the cell surface [57,58,59].

### 2.4. Gateways to Toxicity: Cellular Uptake Pathways

Due to its structural analogy to phosphate, in *E. coli*, arsenate is known to be nonspecifically taken up via phosphate transporters such as the inorganic phosphate [P(i)] transporter (Pit) and phosphate-specific transport (Pst) systems. In contrast, arsenite can enter through aquaglyceroporins such as GlpF, which also mediates glycerol uptake [18,25]. Mutations in the Pit or Pst transporters in *E. coli* confer resistance to arsenate [60,61,62]. Some bacterial species, including *Agrobacterium tumefaciens* and *Streptomyces,* utilize the Pst system for arsenate influx; however, this system exhibits greater specificity for phosphate, thereby reducing arsenate uptake [63,64]. Regarding arsenite, some arsenic-resistant bacteria isolated from contaminated environments exhibit alterations in the GlpF transporter or entirely lack a homologous transporter, as observed in *Thiomonas* sp. 3As and *Herminiimonas arsenicoxydans*, leading to reduce As(III) influx [29,65].

In *B. subtilis*, the Pst system facilitates phosphate uptake but not arsenate, indicating that Pst is not the primary route for arsenate entry in this microorganism [66]. The genome of *B. subtilis* possesses the *pit* (*ykaB*) gene, which encodes a low-affinity phosphate:H^+^ symporter belonging to the Pit family [4]; however, its role in arsenate import remains uncharacterized.

In *E. coli*, arsenite, in the form of arsenious acid As(OH)_3,_ which mimics the structure of glycerol, can be transported via glycerol facilitators such as GlpF [67]. In *B. subtilis*, the *glpF* gene also encodes a glycerol uptake facilitator, and according to a previously unpublished report [68], a GlpF-deficient strain exhibits reduced sensitivity to arsenite, involving this protein in the transport of this ion. Thus, no current study has conclusively identified the arsenic uptake pathway in *B. subtilis* or other species of the genus.

Multiple reports have provided indirect evidence of As uptake in *Bacillus* species. For example, *B. megaterium* and *B. pumilus* strains isolated from an As-contaminated environment, analytical measurements showed 10% cellular arsenic internalization, with the remaining 90% bound to extracellular surface molecules [69]. Additional indirect evidence of As uptake was observed in *B. licheniformis* strains DAS-1 and DAS-2, where high phosphate concentrations in the medium (0.75 mM) exhibited a protective effect, and arsenate removal increased under phosphate-limiting conditions, supporting the involvement of phosphate transporters in arsenate uptake [42]. Intriguingly, in *Bacillus* sp. strain DJ-1, isolated from industrial effluent in Vapi, India, 80% of the accumulated arsenic was found compartmentalized within the cytoplasm. Moreover, whole-cell biosensors developed in *B. subtilis* have demonstrated induction of the *ars* operon in response to both As(III) and As(V), further confirming that this metalloid is internalized by the cell [70,71].

## 3. Molecular Arsenic Resistance Mechanisms in *Bacillus subtilis*

**Figure 1:**
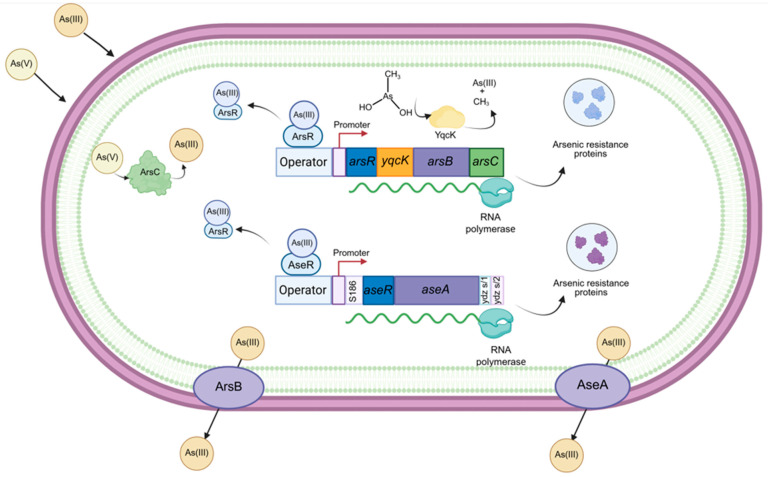
As response genetic system in *B. subtilis*. The genome of this bacterium encodes two As-responsive operons that contribute to metalloid detoxification. The *ars* operon is composed of *arsR* (transcriptional repressor), *yqcK* (organoarsenical lyase), *arsB* (Acr3-type As(III) efflux pump), and *arsC* (As(V) reductase). The *ase* operon is composed of *aseR* (transcriptional repressor) and *aseA* (ArsB-type As(III) efflux pump). Created in BioRender. Valenzuela, L. (2025) https://BioRender.com/qw9l5xk. Image published on 23 September 2025.

*B. subtilis* possesses two chromosomal operons that encode As resistance genes: the *ars* operon, which contributes approximately 50% of the bacterium’s As resistance, and the *ase* operon, accounting for about 40% of its tolerance to this metalloid [72,73] (Figure 1). This section provides a detailed description of the characteristics of the genes that comprise these two operons and their relationship with other As-resistance genes in other *Bacillus* species. It also describes the similarities in amino acid sequences and the structure–function relationships of proteins synthesized according to models generated by experimentation or AlphaFold simulation. Additionally, it includes phylogenetic analyses relating the functional characteristics of each gene product in the *ars* and *ase* operons.

### 3.1. The B. subtilis Ars Operon: A Phage-Derived Genetic Island

The *ars* operon in *B. subtilis* is located within a chromosomal region known as the *sigK* intervening (SKIN) element. This region is named as such because it comprises a 48 kb DNA fragment that disrupts the sigma factor gene *sigK*, creating two truncated genes: *spoIVCB*, which encodes the N-terminal region of Sigma K, and *spoIIIC*, which encodes the C-terminal region. Approximately 3 h after the initiation of sporulation, the site-specific recombinase *spoIVCA* mediates recombination between 5 bp direct repeats, allowing the reconstitution of an intact *sigK* gene. This process excises the skin element from the genome as a circular DNA molecule. Interestingly, this 42 kb element contains 60 ORFs, of which 32 encode proteins homologous to those found in the temperate phage ϕ105 and the defective phage PBSX, suggesting that it is a remnant from a phage DNA sequence [74,75,76].

The *ars* operon is encoded in the chromosomal negative strand (2,657,687 → 2,655,346) (genome version: NC_000964.3) and comprises four genes: *arsR*, *arsI* (*yqcK*), *arsB*, and *arsC*. It is inducible by arsenate, arsenite, and antimonite. A 2.4 kb transcript has been detected in the presence of arsenic, indicating that all four genes are co-transcribed in response to the metalloid. The transcription start site is located 34 nucleotides upstream of the *arsR* start codon. The promoter region includes −10 and −35 elements similar to those recognized by the sigma factor A (σ^A^) [72].

**Figure 2:**
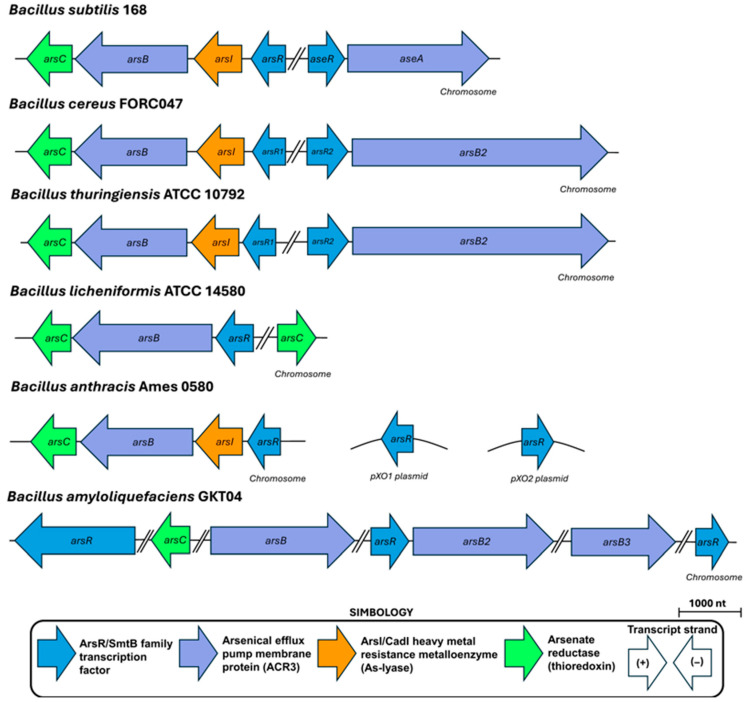
Genetic organization of As response operons in *B. subtilis* and related species within the *Bacillus* genus. Genes are represented by arrows of varying sizes and colors, corresponding to their base pair length and known function. The arrow orientation indicates the transcribed strand. The species name is accompanied by the reference genomes used for the analysis, and the accession numbers for each gene analyzed are listed in Table S1 in the Supplementary Material. Drawing is to scale.

To establish the similarities and genome arrangement of As resistance genes in distinct *Bacillus* species, the genome databases of *B. subtilis*, *B. cereus*, *B. thuringiensis*, *B. licheniformis*, *B. anthracis*, and *B. amyloliquefaciens* were inspected. The accession numbers for these species can be found in Table S1 in the Supplementary Material of this review. The results of these analyses made it apparent that *B. subtilis*, *B. cereus*, and *B. thuringiensis* share similar composition and arrangements of their *ars* operons (Figure 2). In contrast, species like *B. licheniformis*, *B. anthracis*, and *B. amyloliquefaciens* possess *ars* operons that exhibit divergences in number and gene composition, in reference to *B. subtilis* (Figure 2). *B. subtilis* possesses two well-defined operons on its chromosome with As response genes: (i) the four-cistron containing *ars* operon encoded in the negative strand, composed of the transcriptional repressor (*arsR*), the putative arsenite lyase (*arsI*), the arsenite efflux pump (*arsB*), and the arsenate reductase (*arsC*); and (ii) the *ase* bicistronic operon, which is encoded in the positive strand and includes a transcriptional repressor (*aseR*) and an arsenite efflux pump (*aseA*). Similar genetic arrangements are found in *B. cereus* and *B. thuringiensis*. However, *B. anthracis* contains only the *ars* operon located in the negative strand. This operon is complemented by a pair of putative transcriptional repressors belonging to the ArsR/SmtB family encoded in the pXO1 and pXO2 plasmids. *B. licheniformis* has two regions with As response genes. The main operon is similar to the *ars operon* of *B. subtilis* and is located on the negative strand; however, it lacks an As-lyase gene and contains a second As(III) reductase on the positive strand. Interestingly, in *B. amyloliquefaciens,* the As resistance genes are distributed in different regions of its chromosome; furthermore, it possesses three ArsR/SmtB repressors, three As(III) efflux pumps, and one arsenate reductase (Figure 2). Of note, the genes encoded in the *ars* operon of this bacterium share homology with the elements found in the operons of plasmids R773 and R46 of the Gram-negative bacterium *E. coli*. Therefore, some *Bacillus* species exhibit a remarkable diversity in the genetic arrangements and strategies used by *Bacillus* species to cope with arsenic stress.

#### 3.1.1. ArsR: The Metalloid-Sensing Repressor

**Figure 3:**
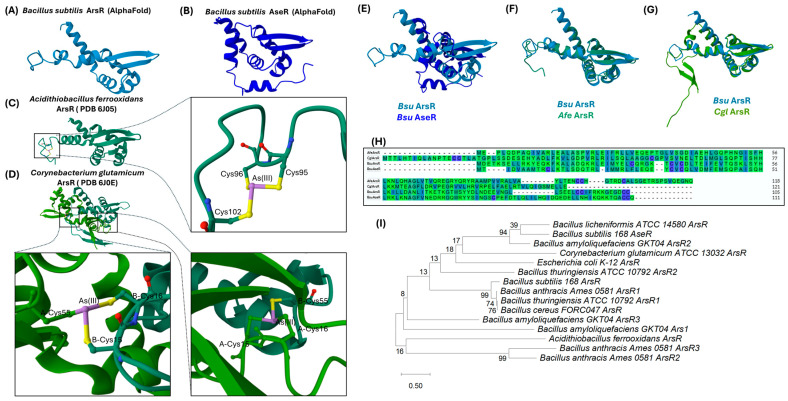
Structural comparison and phylogenetic analyses of different transcriptional repressors of As-responsive operons. (**A**,**B**) AlphaFold-predicted 3D structures of *B. subtilis* (**A**) ArsR (AF-P45949-F1-v4) and (**B**) AseR (AF-P96677-F1-v4). (**C**,**D**) Experimentally resolved 3D structures of (**C**) *A. ferrooxidans* ArsR (PDB: 6J05) and (**D**) *C. glutamicum* ArsR (PDB: 6J0E), with enlarged boxes highlighting cysteine residues critical for As(III) binding. (**E**) Structural overlay between *B. subtilis* ArsR (light blue) and AseR (dark blue). (**F**) Structural overlay of *B. subtilis* ArsR (blue) and *A. ferrooxidans* ArsR (green). (**G**) Structural overlay of *B. subtilis* ArsR (blue) and *C. glutamicum* ArsR (green). (**H**) Amino acid sequence alignment of As-responsive operon repressor proteins; identical residues are shaded with the same color. Alignment is colored in a buried index fashion where darker blue/purple tones indicate deeper burial, while lighter green hues reflect greater exposure. PyMOL (v. 2.3.3.) was used for structure visualization and Clustal Omega (v. 1.2.2.) was used for sequence alignment. (**I**) Evolutionary relationships between various ArsR repressor proteins from *Bacillus* species and other bacteria. The phylogenetic tree was constructed using MEGA12 (v. 12.0.14) software with the amino acid sequences reported in the Table S1 in Supplementary Material. The tree is constructed to scale. Branch lengths are measured by the number of substitutions per site.

In bacteria, the transcriptional regulator protein ArsR, functions as an As(III)-inducible repressor that binds to promoter/operator regions and negatively regulates expression of the entire *ars* operon [77]. In the absence of As, *ars* genes are transcribed at basal low levels, required to keep ArsR-dependent repression. Upon contact with arsenite (As[III]), ArsR loses its DNA-binding affinity, allowing RNA polymerase to increase the transcription rates of the *ars* genes. Structural and functional evidence from *Acidithiobacillus ferrooxidans* and *Corynebacterium glutamicum* has revealed that ArsR binds arsenite at a so-called type I binding site, employing three conserved cysteines to coordinate the metalloid [78,79] (Figure 3C,D).

In *B. subtilis*, the open reading frame of *arsR* consists of 318 bp and encodes a 105 amino acid-long protein with a predicted molecular mass of 12.1473 kDa. *B. subtilis* ArsR (*Bs*ArsR) shares 44% homology with the As repressor from *Staphylococcus* and 31–33% homology with those from *E. coli* encoded on plasmids R773 and R46, respectively [80].

*Bs*ArsR belongs to the ArsR/SmtB family of metalloregulatory proteins, which includes the repressor proteins SmtB, ArsR, and CdaC from diverse bacterial origins. The proteins that belong to the ArsR/SmtB family share structural and functional properties, acting as homodimer repressors that control the expression of resistance genes for Zn, Co, Cd, Pb, Bi, Ni, and metalloids like As and Sb [81]. Furthermore, in its active free-of-metalloid form, these repressors bind to Ars box, A:T rich sequence elements, existing in regulatory regions of the *ars* operons [82]. Upon binding of the metal or metalloid, the repressor protein destabilizes its interaction with DNA, thereby freeing the promoter region and allowing transcription of the genes under the repressor’s control [78,83].

Although *B. subtils* ArsR has not been crystallized, the predicted tridimensional structure derived from AlphaFold analyses (access ID: AF-P45949-F1-v4) [84,85] (Figure 3A) resembles the 3D structures of repressors from *A.ferrooxidans* (PDB: 6J05) [86] (Figure 3C) and *C. glutamicum* (PDB: 6J0E) [86] (Figure 3D), whose 3D structures have been determined through X-ray crystallography, thus evidencing a highly conserved structural similarity (Figure 3F,G). The characteristic structure of ArsR proteins consists of five alpha helices and two beta strands arranged in an α1–α2–α3–α4–β1–β2–α5 fold [86,87,88]. The dimer is formed via a quartet of alpha helices made up of the α2 and α5 helices from each subunit [81]. ArsR proteins contain a helix-turn-helix (HTH) DNA-binding motif, a region that enables the repressor to recognize and bind to the promoter elements of arsenic resistance operons. Regarding the metalloid binding site of ArsR, evidence postulates that it can be located in either the N- or C-terminal domains of each ArsR monomer; and in other cases, the binding site seems to be formed after dimer assembly (Figure 3C,D).

In the ArsR protein encoded in the *E. coli* plasmid R773, the coordination for As(III) binding relies on the residues Cys32, Cys34, and Cys37 located in the α3 helix of the N-terminal end (Type I site) [83,89]. With respect to *A. ferrooxidans,* the metalloid binding to ArsR is coordinated by the residues Cys95, Cys96, and Cys102, located in the C-terminal α5 helix (Figure 3C, enlarged) [88]. In contrast, the As-binding site of *C. glutamicum*’s ArsR is established by residues Cys15 and Cys16 existing in one of the monomers and Cys55 in the partner protein (Figure 3D, enlarged) [86]. In *B. subtilis*, the repressor ArsR contains two potential binding sites: one in the α3 helix (Cys38, Cys40, and Cys32) and another in the α5 helix (Cys93, Cys94, Cys104, Cys105) [68] (Figure 3H).

To further extend the structural conservation analysis of *Bacillus* ArsR proteins, a phylogenetic tree was constructed using the maximum likelihood method, with MEGA12 software (v. 12.0.14) set to a bootstrap value of 1000 replicates. This tree compared the amino acid sequences of ArsR proteins encoded by the genes indicated in Figure 2, as well as ArsR proteins from other bacteria. Figure 3I reveals high similarity among the ArsR repressors of *B. subtilis*, *B. anthracis*, *B. thuringiensis*, and *B. cereus*. These species are placed within the same clade, with a high bootstrap value. In contrast, ArsR from *B. subtilis* was found to significantly diverge from its counterparts in *E. coli* and *C. glutamicum*; and such divergence was even greater with ArsR from *A. ferrooxidans*. Therefore, despite significant differences, ArsR homologues diverging from typical *Bacillus* species seem to maintain their ability to bind arsenite and activate an As resistance response.

The expression of arsenic-responsive genes is controlled by a regulatory region composed of two *cis-acting* elements, a promoter dependent on the σ^A^ transcription factor, and an operator binding site for the ArsR homodimer. The ArsR operator does not directly overlap with the RNA polymerase binding site but is located slightly upstream of this promoter. In *E. coli*, the repressor dimer binds to an imperfect inverted repeat DNA sequence spanning from nucleotide −64 to −40 relative to the transcription start site of the *ars* operon [82]. In *B. subtilis*, the transcription start site of the *ars* operon was located 34 nucleotides upstream of the *arsR* start codon, and the putative RNA polymerase binding sites at positions −35 (TTGCAT) and −10 (TATAAT) were also identified in the gene’s promoter region [72]. The ArsR repressor protein binding site is suggested to be an 8 bp inverted repeat located 2 bp upstream of the −35 region [72].

#### 3.1.2. ArsI: The Organoarsenical Lyase

The 441 bp long *yqcK* gene of *B. subtilis*, also known as *arsI*, codes for a protein with arsenic lyase activity (ArsI, from As-induced), capable of breaking the carbon–arsenic (C–As) bonds of organoarsenic compounds. ArsI is composed of 146 amino acids with a predicted molecular mass of ~16.5 kDa and is catalytically active in its monomeric form [75,90]. *arsI* genes are widely distributed among aerobic bacteria and play a fundamental role in degrading organoarsenical compounds in the environment [91]. ArsI activity directly influences the mobility and toxicity of arsenic in ecosystems [92]. ArsI belongs to a class of non-heme ferrous iron [Fe(II)]-dependent dioxygenases, which use molecular oxygen to catalyze the cleavage of the C–As bond [92]. This activity has been found to be essential for the detoxification of organoarsenical compounds and for *B. subtilis*’ adaptation to arsenic-contaminated environments, underscoring its role in the biogeochemical cycle of this element [91].

Organoarsenical compounds such as monosodium methylarsonate [MAs(V)] and roxarsone [Rox(V)] have been widely used as herbicides and animal growth promoters. MAs(V) is primarily applied as a post-emergent herbicide for selective weed control. Although considered less toxic than inorganic arsenic species, it can undergo environmental degradation to yield the more toxic As(III) [91,93]. Roxarsone, in turn, is commonly added to poultry and livestock feed due to its anticoccidial activity. It improves efficiency and accelerates growth. However, arsenic-rich manure from treated animals—often used as fertilizer—contains 70–90% water-soluble arsenic, which can leach into soil and water systems [94]. Degradation of these compounds by ArsI yields As(III), which can contaminate crops and drinking water sources [95].

ArsI catalyzes the demethylation of methylarsonite [MAs(III)] to As(III) and the dearomatization of aromatic arsenicals like Rox(III) [91]. Its mechanism involves Fe(II) binding to the active site, followed by interaction with the organoarsenic substrate. Molecular oxygen then binds to Fe(II) and is inserted into the C–As bond, leading to its cleavage and the release of As(III). Structural studies have identified Gln8, His65, and Glu117 in *Thermomonospora curvata* ArsI (corresponding to His5, His62, and Glu115 in *Bacillus* ArsI) as critical for metal binding. Additionally, Cys98 and Cys99 (Cys96 and Cys97 in *Bacillus*) form the substrate-binding site [93] (Figure 3H). A loop-gating mechanism has been proposed, in which a structural loop in ArsI regulates substrate and product access [92].

Mutation of key residues, such as Lys105 in *T. curvata*, to alanine, arginine, or glutamate leads to a loss of catalytic activity and decreased Fe(II) affinity, suggesting that Lys105 is involved in dioxygen activation [95]. Furthermore, chemical modification of cysteine, histidine, and glutamic/aspartic acid residues inhibits ArsI activity, confirming their catalytic importance. Mutation of Cys96 and Cys97 to serine abolishes substrate binding and enzymatic activity [93]. Tyr38, Gln103, and Lys105 also interact with Fe(II) through water molecules and are essential for catalysis [92].

**Figure 4:**
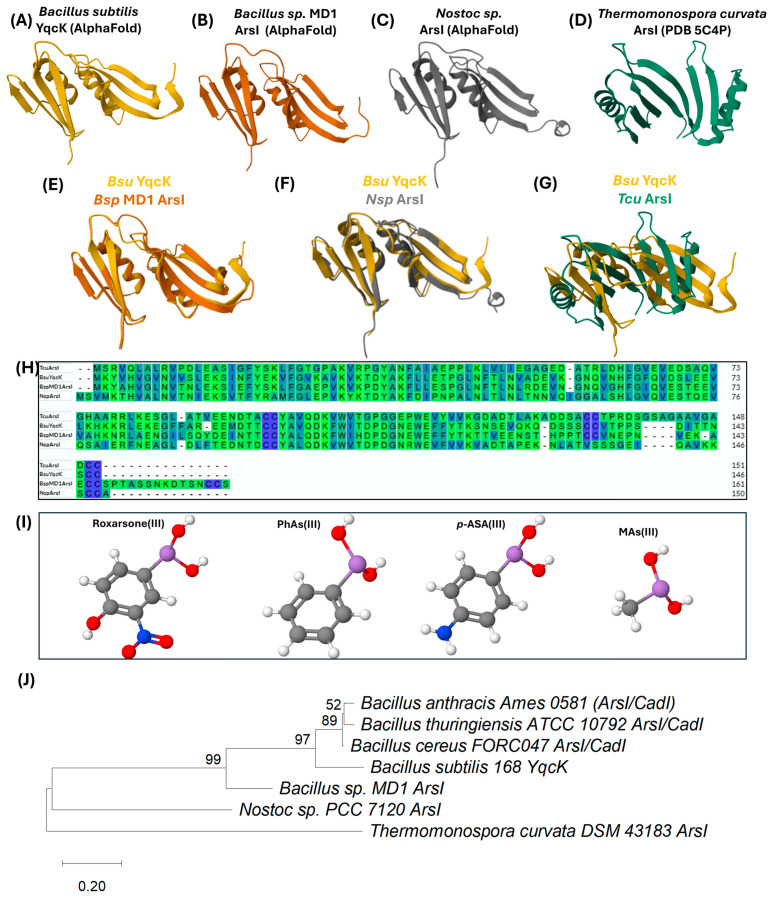
Structural comparison and phylogenetic analysis of bacterial As-lyase proteins. (**A**–**C**) AlphaFold-predicted 3D structures of (**A**) *B. subtilis* YqcK, (**B**) *Bacillus* sp. MD1 ArsI, and (**C**) *Nostoc* sp. ArsI. (**D**) Experimentally resolved 3D structure of *T. curvata* ArsI (PDB: 5C4P). (**E**) Structural comparison between *B. subtilis* YqcK (yellow) and *Bacillus* sp. MD1 ArsI (orange). (**F**) Structural comparison between *B. subtilis* YqcK (yellow) and Nostoc sp. ArsI (gray). (**G**) Structural comparison between *B. subtilis* YqcK (yellow) and *T. curvata* ArsI (green). (**H**) Amino acid sequence alignment of As-lyase proteins; identical residues are shaded with the same color. Alignment is colored in a buried index fashion where darker blue/purple tones indicate deeper burial, while lighter green hues reflect greater exposure. PyMOL (v. 2.3.3.) was used for structure visualization and Clustal Omega (v. 1.2.2.) for sequence alignment. (**I**) Structures of organoarsenical compounds: roxarsone (III), phenylarsenite (III), p-arsanilic acid (III), and methylarsenite (III), which are known substrates of the ArsI protein from *T. curvata*. Molecular structures of substrates were rendered using MolView (URL: https://molview.org; accessed on 28 July 2025). (**J**) Evolutionary relationships between various As-lyase proteins from *Bacillus* species and other bacteria. The phylogenetic tree was constructed using MEGA12 (v. 12.0.14) software with the amino acid sequences reported in Table S1 in the Supplementary Material. The tree is constructed to scale. Branch lengths are measured by the number of substitutions per site.

Isothermal titration calorimetry (ITC) studies have shown that Fe(II) binds to ArsI with a dissociation constant (Kd) of approximately 4 µM. In contrast, As(III), the reaction product, does not bind significantly to ArsI. However, MAs(III) and phenylarsenite [PhAs(III)], a Rox(III) analog, bind the enzyme with Kd values of 0.3 µM and 0.6 nM, respectively, indicating a higher affinity of ArsI for Rox(III). Substrate binding results in the quenching of ArsI’s tryptophan fluorescence, enabling quantification of its affinity for different compounds using intrinsic protein fluorescence quenching techniques [93]. Rox(III) degradation by ArsI produces As(III) and 2-nitrohydroquinone, confirming that the enzyme acts as a dioxygenase that catalyzes the rupture of the C-As bond by incorporating one oxygen atom from O_2_ into the carbon and another into the arsenic [91,93]. A graphical representation of the organoarsenical substrates processed by ArsI from *T. curvata* is shown in Figure 4H.

Additionally, the cyanobacterium *Nostoc* sp. has been found to possess an ArsI enzyme (NsArsI) capable of demethylating MAs(III) to As(III). In this organism, NsArsI mainly exists in a trimeric state. Heterologous expression of *NsarsI* in *Escherichia coli* confers resistance to MAs(III), suggesting its potential application in environmental bioremediation [96]. A comparative analysis of the primary and tertiary structures (Figure 4E,H) of As-lyases from different bacteria revealed that YqcK from *B. subtilis* (access ID: AF-P45945-F1-v4) [84,85] (Figure 4A) exhibits a high degree of similarity with the ArsI enzyme from *Bacillus* sp. MD1 (access ID: AF-A0A059WI14-F1-v4) [84,85] (Figure 4B), an environmental strain isolated from a golf course in Florida (Figure 4E) [91]. Interestingly, high structural similarity was also observed between AlphaFold models of *B. subtilis* YqcK and *Nostoc* sp. (access ID: AF-A0A1Z4KSY1-F1-v4) [84,85] (Figure 4C,F) ArsI. In contrast, although the crystal structure of *T. curvata* ArsI has been used as a reference for predicting other As-lyase topologies [93], it only shows moderate structural homology with *B. subtilis* YqcK (Figure 4G).

In agreement with the comparative structural study of As-lyases described above, a phylogenetic analysis based on amino acid sequences revealed that YqcK from *B. subtilis* is more closely related to ArsI enzymes from *Bacillus* sp. MD1 (84% similarity) and *Nostoc* sp. (81% Similarity) than to ArsI from *T. curvata* (41% similarity). The latter is the only crystallized As-lyase recorded in the literature; consequently, the majority of inferences regarding As-lyase functions have been derived this protein. These findings highlight the need for structural studies of YqcK from *B. subtilis* and other ArsI enzymes from *B. anthracis*, *B. thuringiensis*, and *B. cereus*, as they are all phylogenetically related (Figure 4J). These studies will refine our understanding of the molecular mechanisms of resistance to organoarsenical compounds in the genus *Bacillus*.

#### 3.1.3. ArsB: The Acr3-Type Efflux Pump

**Figure 5:**
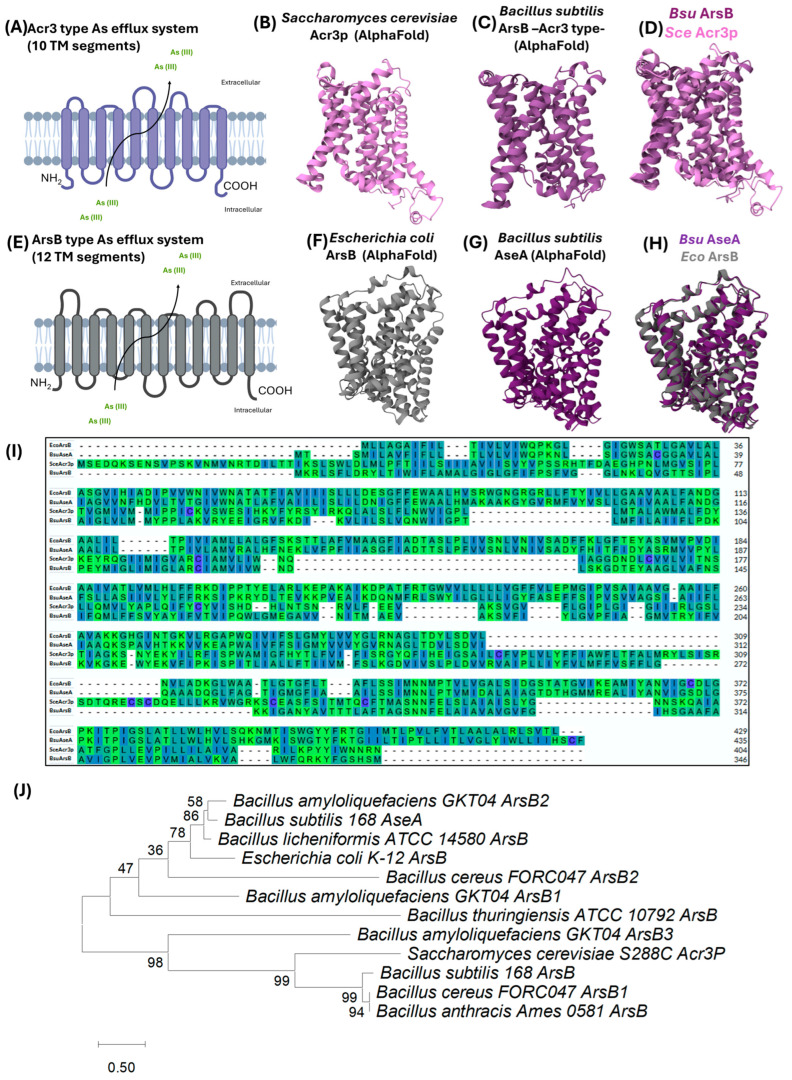
Transmembrane domains, sequence, structural comparative, and phylogenetic analysis of As(III) efflux system proteins. (**A**) Topological arrangement of Acr3-type As(III) efflux transporters containing 10 transmembrane segments. (**B**,**C**) AlphaFold-predicted 3D structures of Acr3-type proteins: (**B**) *S. cerevisiae* Acr3p and (**C**) *B. subtilis* ArsB. (**D**) Structural comparison between *B. subtilis* ArsB (magenta) and *S. cerevisiae* Acr3p (light purple). (**E**) Topological arrangement of ArsB-type As(III) efflux transporters containing 12 transmembrane segments. (**F**,**G**) AlphaFold-predicted 3D structures of ArsB-type proteins: (**F**) *Escherichia coli* ArsB and (**G**) *B. subtilis* AseA. (**H**) Structural comparison between *E. coli* ArsB (gray) and *B. subtilis* AseA (dark purple). (**I**) Amino acid sequence alignment of As(III) efflux system proteins; identical residues are shaded with the same color. The alignment is colored by burial index (darker blue/purple: buried residues; lighter green: solvent-exposed residues). PyMOL (v. 2.3.3.) was used for structure visualization, and Clustal Omega (v. 1.2.2.)was used for sequence alignment. (**J**) Evolutionary relationships between various As efflux pump proteins from *Bacillus* species and other bacteria. The phylogenetic tree was constructed using MEGA12 (v. 12.0.14) software with the amino acid sequences reported in Table S1 in the the Supplementary Material. The tree is constructed to scale. Branch lengths are measured by the number of substitutions per site.

*arsB*, the third gene found in the *ars* operon of *B. subtilis,* is composed of 1041 bp. It encodes a 346 amino acid protein with a predicted molecular mass of ~38.1 kDa [90]; this protein has been involved in the efflux of arsenite from the cell, a highly conserved As resistance mechanism in bacteria. Although referred to as *arsB* (*yqcL*) in *B. subtilis*, this gene differs significantly from the *arsB* genes from *E. coli* and *S. aureus*, as it shares only 24% sequence identity with the latter [72,97]. Indeed, *Bs*ArsB is more closely related to membrane proteins that belong to the Arsenical Resistance-3 (ACR3) family, as is depicted in the phylogenetic tree of Figure 5J. Both *arsB* and *acr3* encode membrane-bound proteins involved in arsenic resistance in prokaryotes but differ in transport mechanism, substrate specificity, and phylogenetic distribution [25].

While ArsB is restricted to prokaryotes, Acr3 has a broader distribution, being found in bacteria, archaea, fungi, and even in some plants. The prevalence of *arsB* and *acr3* varies by taxonomic group: *acr3* predominates in Proteobacteria, Firmicutes, Actinobacteria, and Asgardarchaeota, while *arsB* is more common in Alphaproteobacteria, Gammaproteobacteria, and Campylobacterota [25,98]. Of note, the product of the *arsB/yqcL* gene (ID access: AF-P45946-F1-v4) [84,85] located in the SKIN element of *B. subtilis* shows greater homology to the Acr3p transporter of *Saccharomyces cerevisiae* [84,85] (access ID: AF-Q06598-F1-v4) [84,85] than to the *E. coli arsB* transporter [84,85] (access ID: AF-P0AB93-F1-v4) [84,85] (Figure 5B–D,I,J).

The well-characterized *E. coli* ArsB transporter has been shown to contain 12 transmembrane helices (Figure 5E) and is coupled to a dimeric ATPase encoded by *arsA*. In contrast, Acr3-type transporters, despite their widespread presence across all domains of life, have not yet been crystallized [28]. Genetic studies using translational fusions to alkaline phosphatase and green fluorescent protein have shown that the *B. subtilis* Acr3 protein comprises 10 transmembrane helices, with both the N- and C-termini facing the cytoplasm [97] (Figure 5A).

The Acr3-type transporters are powered by a proton motive force (H^+^ gradient) to export arsenite; therefore, they function as antiporters. Their primary substrate is arsenite (As[III]) (Figure 5A,E), although they can also transport antimonite (Sb[III]) [99].

A recent study elucidated the transport mechanism of these antiporters in *E. coli* BL21 (Δ*arsB*) expressing Acr3Bs mutants. To this end, the authors employed AlphaFold2 structural modeling, evolutionary conservation analysis, conserved motif identification via ConSurf and WebLogo, Western blotting, and microscale thermophoresis (MST) to measure arsenite-binding affinity. Results from these analyses revealed that Acr3 antiporters of the BART superfamily adopt a folding pattern typical of the Na^+^/H^+^ antiporter NhaA [100]. The predicted structure of *B. subtilis* ArsB (Acr3Bs) includes 10 transmembrane helices (TM1–TM10) arranged in two topologically inverted repeats (TM1–TM5 and TM6–TM10). In the central domain, discontinuous helices TM4 and TM9 interact to form an X-shaped structure (Figure 5C). Three conserved motifs have been identified: Motif A (GxARCxA) in the TM4 loop, Motif C (NNFE) in TM9, and Motif B (LNSxxQ) in TM5; these form the active binding pocket. Motive A is located in the N-terminal of Acr3Bs; furthermore, it has been shown that the residues G115, R118, and C119 are required for the efficient transport of arsenite [100]. Residues N292 and E295 in Motif C form hydrogen bonds with arsenite, directly contributing to transport. Motif B may be involved in protein protonation/deprotonation; residues R118 (TM4) and E322 (TM10), in this motif, interact with N292 (TM9) to stabilize the X-helix structure [100].

Notably, although the *ars* operon can be induced by Sb (III), studies have shown that Acr3Bs expression in *E. coli* confers only marginal resistance to Sb (III), while it significantly enhances resistance to As (III) [72,100]. Results derived from a comparative amino acid alignment between ArsB from *B. subtilis*, Acr3p from *S. cerevisiae,* and ArsB proteins from *E. coli* and *S. aureus* show the conserved motifs already mentioned (Figure 5I).

#### 3.1.4. ArsC: The Arsenate Reductase

*arsC* (*yqcM*), the fourth gene in the *ars* operon of *B. subtilis,* is 420 bp long and encodes an arsenate reductase of 139 amino acids with a molecular weight of 15.4 kDa [90], whose crystal structure has been resolved [101]. Arsenate taken up through phosphate transporters can be converted and detoxified in the cytoplasm initially by ArsC, which catalyzes the reduction of arsenate (As[V]) to arsenite (As[III]), a key step in arsenic detoxification. Once reduced, arsenite can be exported by efflux systems such as ArsB and Acr3 or methylated by enzymes like arsenite-S-adenosylmethionine methyltransferase (ArsM) in certain microorganisms [19,27,102,103].

**Figure 6:**
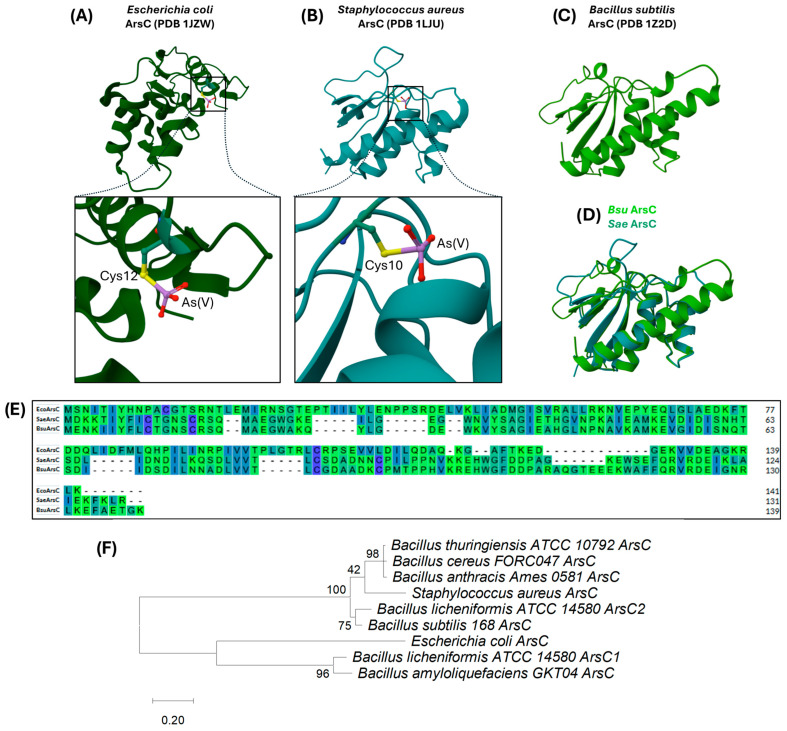
Structural comparative and phylogenetic analysis of different As (V) reductase proteins. (**A**–**C**) Experimentally resolved 3D structures of (**A**) *E.coli* ArsC (PDB: 1JZW); (**B**) *S. aureus* ArsC (PDB:1LJU) and (**C**) *B. subtilis* ArsC (PDB: 1Z2D). Enlarged boxes in (**B**,**C**) highlight cysteine residues critical for As(V) binding. (**D**) Structural overlay between *B. subtilis* ArsC (green) and *S. aureus* AseR (blue). (**E**) Amino acid sequence alignment of As(V) reductase proteins; identical residues are shaded with the same color. The alignment is colored by burial index (darker blue/purple: buried residues; lighter green: solvent-exposed residues). PyMOL (v. 2.3.3.) was used for structure visualization, and Clustal Omega (v. 1.2.2.) was used for sequence alignment. (**F**) Evolutionary relationships between various arsenate reductases from *Bacillus* species and other bacteria. The phylogenetic tree was constructed using MEGA12 (v. 12.0.14) software with the amino acid sequences reported in the Table S1 in the Supplementary Material. The tree is constructed to scale. Branch lengths are measured by the number of substitutions per site.

It has been suggested that bacterial and archaeal *arsC* gene sequences reflect a common ancient origin for arsenate reductases, possibly emerging in the reducing atmosphere of early Earth. Initially, enzymes detoxifying As(III) may have been critical, while the post-Great Oxidation Event environment, richer in As(V), drove the evolution of arsenate-reducing enzymes [102]. The ArsC reaction mechanism involves the direct binding of arsenate at the enzyme’s active site, where it is reduced to arsenite. In *E. coli*, ArsC contains a single cysteine residue (Cys-12) in the active site, flanked by an arginine triad (Figure 6A enlargement). In contrast, in *S. aureus*, the enzyme has three additional cysteine residues, including Cys-10, Cys-15, and Cys-90 (Figure 6B enlargement), indicating structural and mechanistic diversity among bacterial arsenate reductases. Interestingly, the ArsC protein from *B. subtilis* exhibits significant sequence and structural similarities to the ArsC protein from *S. aureus* (Figure 6D,E). In fact, a phylogenetic analysis of several ArsC proteins of the *Bacillus* species, including *B. subtilis*, *B. thuringiensis*, *B. cereus*, *B. anthracis,* and *B. licheniformis* ArsC2, showed that they are all phylogenetically related to ArsC from *S. aureus*, as they are found in the same clade. However, they diverge from the ArsC from *E. coli*. In fact, *E. coli* ArsC is related to ArsC1 from *B. licheniformis* and ArsC from *B. amyloliquefaciens,* evidencing the genetic diversity among *Bacillus* species.

### 3.2. The Ase Operon: A Chromosomal Arsenic Resistance System

*B. subtilis* possesses an additional arsenic resistance operon located in the genomic region 579,354 (+) → 581,741 (+) (Figure 2), which is distant from the *ars* operon embedded within the SKIN element and transcribed from the negative strand. The *ase* operon is composed of two genes: *aseR* and *aseA*; and two pseudogenes, *ydzS/1*, and *ydzS/*2 (Figure 2, pseudogenes omitted). The *aseR* gene encodes a regulatory protein of the operon (Figure 3B), and *aseA* encodes an efflux pump for As (III) (Figure 5G), while the function of *ydzS/1* and *ydzS/2* is currently unknown. This operon lacks an arsenate reductase, leading some authors to suggest that the *ase* operon could be more ancient in *Bacillus*, possibly arising during the conditions of a reducing atmosphere. In contrast, the *ars* operon is believed to have been integrated into the genome of *B. subtilis* via horizontal gene transfer during evolution into the *sigK* gene, as the SKIN element exhibits characteristics of a prophage [73,74]. However, it has been demonstrated that the *ase* operon contributes to the arsenic (III) resistance conferred by the *ars* operon [73].

#### 3.2.1. AseR: A Second Layer of Regulation

The 336 bp long open reading frame of *aseR* codes for a 111-amino acid protein with a predicted molecular mass of 12.9 kDa [90]. AseR is a transcriptional repressor belonging to the ArsR-SmtB family, although it shares only approximately 27% homology with ArsR from *B. subtilis* [73,97] (Figure 3B,E,H). In fact, a structural comparison of the AseR (access ID: AF-P96677-F1-v4) [84,85] and ArsR (access ID: AF-P45949-F1-v4) [84,85] 3D models does not reveal the same degree of similarity as that exhibited between the ArsR model from *B. subtilis* and its homolog in *A. ferrooxidans*, which is more closely related to *B. subtilis* phylogenetically (Figure 3H). Despite the low bootstrap value, the phylogenetic analysis in Figure 3I indicates that AseR is a variant of ArsR from *B. subtilis,* and therefore, a more recent version of this transcriptional repressor; however, this cannot be unambiguously assessed due to the mobility of the SKIN element. The SKIN element contains the *ars* operon genes and is known to have originated from an ancestral template phage [74]. While both proteins regulate the expression of As resistance genes in *B. subtilis,* they differ in specificity and response mechanisms to As.

Like ArsR, AseR forms a homodimer that interacts with DNA through its helix-turn-helix (HTH) domain. Although no crystal structure of AseR is currently available, the DNA-binding domain has been mapped to helices α3 and α4 (Figure 3). The critical amino acids for metalloid binding—Cys33, Cys35, and Asp39—are also located in helix α3, forming a triangular geometry as that described for ArsR [78] (Figure 3). In AseR, arsenite binds to the thiol groups of cysteines in the α3 helix, inducing a conformational change that decreases DNA affinity. While *in vitro* assays showed that AseR can also bind zinc at the same sites, this does not trigger release from DNA, suggesting that the triangular geometry is key to specificity.

It has been shown that AseR binds to the inverted repeat sequence TATATAACGATTTGCTTATATA located in the operon promoter region of the *ase* operon. This inverted repeat is a conserved binding site for transcriptional regulators involved in arsenic resistance [78].

#### 3.2.2. AseA: An ArsB-Type Efflux Pump

The *aseA* gene, 1308 bp long, is the second gene in the *ase* operon (Figure 2) whose transcription is regulated by the AseR repressor [78]. The protein coded is 435 amino acids long and possesses a predicted molecular mass of 47.1 kDa. AseA functions as an As(III) efflux pump contributing to As(III) but not As(V) resistance as it can only export arsenite outside the cells [73]. Deletion of the *aseA* gene in a genetic background lacking the *ars* operon increased *B. subtilis* sensitivity to arsenite; however, the single deletion of *aseA* from the WT parental strain had a minimal impact on such resistance due to the presence of the *ars* operon [73]. In *B. subtilis* strains lacking the SKIN element, the AseA protein may be solely responsible for arsenic efflux [104].

Evidence derived from comparative structural analyses based on AlphaFold simulations has revealed that AseA from *B. subtilis* (access ID: AF-A0A164VUH8-F1-v4) [84,85] shares significant similarity with ArsB from *E. coli*. Indeed, both proteins belong to the ArsB permease family of transporters, which are characterized by containing twelve transmembrane passages (Figure 5B). In contrast, ArsB from *B. subtilis* has been shown to belong to the ACR3 family; however, proteins from this family exhibit only ten transmembrane passages (Figure 5A). Of note, both AseA and ArsB are broadly distributed across *Bacillus* species (Figure 5J). A phylogenetic analysis confirmed that AseA from *B. subtilis* is included in the same clade as ArsB from *E. coli* and *B. licheniformis,* as well as ArsB2 from *B. amyloliquefaciens.* Interestingly, *B. amyloliquefaciens*, a common biocontrol agent, contains *arsB*, encoding a third enzyme that facilitates the expulsion of As(III), which is more related to the ArsB permease family (Figure 5J). In conjunction, these observations highlight the diversification of proteins produced by environmental bacteria of the genus *Bacillus* specialized in the expulsion of arsenite.

### 3.3. Beyond Core Operons: Additional Genetic Determinants

In addition to the genes described in the *ars* and *ase* operons, additional determinants of As resistance have been identified in bacteria of the genus *Bacillus*, including *aioAB*, *aioXRS*, *arrAB*, *aioS*, and *arsD*; however, *arsH* and *arsM* detected in other genera are absent from the *Bacillus* species.

The *aioAB* genes encode subunits of an arsenite oxidase, which can be found in some As resistance operons in bacteria in conjunction with *aioR* and *aioS* genes. The structure of the AioB subunit possesses a Rieske [2Fe-2S] domain that enables electron transfer. This enzyme can convert arsenite into the less toxic arsenate, allowing As(III) to serve as an electron donor for anaerobic respiration. In *Bacillus* sp. strain IIIJ3-1, the product of the *aioB* gene has been shown to oxidize arsenic under aerobic conditions with kinetic values of Km = 2.8 mM and Vmax = 0.2 mM h^−1^. However, despite its functional similarity, a phylogenetic analysis revealed a distant relationship of this gene to an oxidase from *Listeria monocytogenes* [56].

The *aioXSRS* operon, involved in arsenite oxidation, was detected in the isolate *B. firmus* L-148 [57]. The cistron *aioR*, codes for a regulator of the histidine kinase sensor, which is coded by the second cistron, *aioS*. The latter detects As (III) in the environment, autophosphorylates at a histidine residue, and transfers the phosphate group to AioR, which then binds the operon’s promoter region [57].

The *arrAB* operon from the microorganism *B. selenitireducens* has been involved in As resistance. Consistently, *arrA* and *arrB* code for the large and small subunits of a respiratory arsenate reductase, respectively. This dimeric enzyme uses arsenate as the final electron acceptor in the respiratory chain under anaerobic conditions, facilitating energy generation. The *arrAB* genes in *B. selenitireducens* are part of an operon that also includes *arrD*, a chaperone protein, and *arrC*, a membrane subunit involved in anchoring and electron transfer [105].

The *arsD* gene is commonly found in five-gene *ars* operons, adjacent to *arsA*, suggesting a co-evolutionary acquisition. *arsD* can be located on plasmids or in chromosomes in both bacteria and archaea. Notable examples from non-*Bacillus* species include *E. coli* (plasmid R773 *arsRDABC* operon), *Klebsiella oxytoca* (plasmid pMH12), *Acidiphilium multivorum* (plasmid pKW301), *Salmonella typhimurium* (plasmid R46), *Halobacterium* sp. NRC-1 (plasmid pNRC100 with *arsADRC*), and *Sinorhizobium* sp. As4 [106,107]. In *Bacillus*, *arsD* has been identified in some operons, including *Bacillus* sp. UWC [108], *Bacillus* sp. CDB3 [109], *Bacillus* sp. PVR YHB 1-1 [110], *B. paralicheniformis ZAP17*, and *B. altitudinis ZAP62* [111]. ArsD plays a bifunctional role, as a transcriptional repressor and a metallochaperone, for heavy metal regulation and detoxification [112]. Firstly, it acts as a repressor regulating *ars* operon expression [113]. Furthermore, together with ArsR, ArsD modulates gene expression, where ArsR sets the basal level and ArsD potentiates expression of the *ars* operon. Of note, deletion of *arsD* results in the overexpression of *arsA*, *arsB*, and *arsC* [82]. Additionally, evidence has shown that ArsD facilitates the transfer of As(III) to the ArsA ATPase, a subunit of the ArsAB efflux pump. This interaction increases ArsA’s affinity for As(III), thus enhancing resistance to environmental arsenite [106].

Structural analysis revealed that ArsD has neighboring cysteine pairs (Cys12-Cys13, Cys112-Cys113, Cys119-Cys120), which are essential for metal binding. Although Cys12-Cys13 is the only conserved pair across all ArsD homologs, each pair may form a separate metalloid binding site. Residues Cys12, Cys13, and Cys18 are directly involved in the As(III) transfer to ArsA. This interaction occurs at cysteine-rich metalloid-binding sites. *In silico* models suggest that the α1 helix of ArsD and its metalloid binding site facilitate interaction with ArsA [112]. The transfer of As(III) requires ATP hydrolysis and a transient conformation of ArsA during its catalytic cycle [106].

Another gene found in the *ars* operons is *arsN*, which encodes a putative acetyltransferase, though its function is not fully understood. This gene has been identified in bacterial genomes of *Shingomonas* sp. SKA58, *Rhizobium leguminosarum* bv. viciae 3841, *Rhodobacter sphaeroides* ATCC17025, *Burkholderia* sp. 383, *Burkholderia vietnamiensis* G4, and *Burkholderia multivorans* ATCC 17616. Experimental evidence suggests that *arsN* confers arsenate resistance via a mechanism that requires other As detoxification components like *arsC* or *arsD* [114].

In the context of the recognition and degradation of organoarsenical compounds, as mentioned above, *B. subtilis* possesses the *arsI* gene (*yqcK*), encoding a putative carbon–arsenic lyase that could degrade organoarsenicals to inorganic arsenic [91]. However, genes like *arsH* and *arsM*, involved in organoarsenical degradation, are absent in this microorganism. ArsH, on the one hand, belongs to the FMN- and NADPH-dependent oxidoreductase superfamily that can operate over organoarsenicals, highlighting its role in redox reactions vital for survival in As-contaminated environments [115]. ArsH oxidizes trivalent organoarsenicals into less toxic pentavalent species. The *arsH* gene has been found in *P. putida* and plasmids of *Serratia marcescens* and *Sinorhizobium* sp., suggesting broad distribution and evolutionary relevance for adaptation to arsenic-rich environments [115]. Heterologous expression studies showed that introducing *arsH* from *P. putida* into *E. coli* grants resistance to MAs(III), phenylarsenite (PhAs(III)), and Rox(III). Interestingly, the *yhdA* gene of *B. subtilis* encodes an NADPH-dependent FMN oxidoreductase that shares 25.4% homology with ArsH (Table 1), and although its involvement in chromate detoxification has been described [116], it remains to be clarified whether it could have a role in the detoxification of organoarsenical species.

On the other hand, ArsM catalyzes the transfer of a methyl group from S-adenosylmethionine (SAM) to arsenite, producing monomethylarsonic acid (MMA), dimethylarsinic acid (DMA), and sometimes trimethylarsine (TMA). This methylation process is key to arsenic biotransformation, impacting both detoxification and environmental mobility of As [21]. ArsM’s catalytic mechanism involves the binding of As(III) and SAM to the protein, followed by thiol and methyl group transfers. Conserved cysteine residues at the active site are essential for As binding and maintaining its reduced state [21]. *arsM* genes have been identified in distinct bacteria, including *Rhodopseudomonas palustris*, *Pseudomonas* spp., *Halobacterium* sp. NRC-1, *Streptomyces* sp., *Amycolatopsis mediterranei*, *Sphaerobacter thermophilus*, *Geobacillus kaustophilus*, *Rubrivivax benzoatilyticus*, and *Conexibacter woesei* [117]. *arsM* has not been detected in *Bacillus* species, though some studies suggest that these bacteria can volatilize As, hinting at alternative enzymes or mechanisms [21,117]. Although no enzymes capable of methylating arsenic (As) have been identified in *B. subtilis*, genome analysis revealed an open reading frame, *ydaC*, which encodes a putative methyltransferase that shares nearly 30% homology with ArsM (Table 1). This opens the possibility of further researching the functions of uncharacterized proteins that could be involved in arsenic resistance.

A key question about organoarsenicals is, how do they enter and exit the cell? Membrane proteins such as ArsJ, ArsP, and ArsK have been identified in some bacteria, with roles in exporting organic As compounds, while uptake mechanisms remain less characterized. Aquaglyceroporins and sugar permeases may help import certain organoarsenical species [21]. The ArsJ permease in *P. aeruginosa* DK2 might export pentavalent organoarsenicals like 1-arseno-3-phosphoglycerate [103,118], while ArsP and ArsK proteins can export trivalent organoarsenicals such as methylarsenite (MAsIII) and roxarsone (RoxIII). *arsP* genes have been detected in *Campylobacter jejuni*, *Cupriavidus metallidurans*, and *Shewanella putrefaciens* [119]. *arsK* genes are conserved in *Agrobacterium*, *Rhizobium*, *Ensifer*, *Mesorhizobium*, *Pseudochrobactrum*, and *Sinorhizobium* [103,115,120]. So far, none of these genes (*arsP*, *arsJ*, or *arsK*) have been detected in arsenic resistance operons of *Bacillus* species, leaving the question of whether specific permeases for organic arsenic compounds exist in this genus open. One possibility is that the *ycgR* gene of *B. subtilis* could function as a transporter of organic species of As. Its product is 23% similar to ArsP, and it has been identified as a membrane protein (Table 1). However, its function remains to be characterized experimentally.

Some genes like *arsN*, *arsO*, and *arsT* have been found in As resistance operons, but their functions remain poorly understood. The *arsN* gene encodes a putative acetyltransferase and has been found in *Shingomonas* sp. SKA58, *Rhizobium leguminosarum* bv. viciae 3841, *Rhodobacter sphaeroides* ATCC17025, *Burkholderia* sp. 383, *Burkholderia vietnamiensis* G4, and *Burkholderia multivorans* ATCC 17616. Experimental evidence suggests that *arsN* confers arsenate resistance via a mechanism that requires other As detoxification components such as *arsC* or *arsD* [114]. The *arsO* gene encodes a putative flavin-binding monooxygenase, whereas *arsT* encodes a thioredoxin reductase [103,121]. The *arsT* gene has been identified in the strain *Bacillus* sp. CDB3, which contains an eight-gene operon (*arsRYCDATorf7orf8*) [122]. Although no homolog has been found for *arsN* in *B. subtilis*, it is known experimentally that CzcO is involved in cation transport and is 27% similar to ArsO. YumC, meanwhile, has been characterized as a ferredo-xin/flavodoxin-NAD(P) oxidoreductase and bears a modest similarity to ArsT. Table 1 lists Ars resistance homologs from *B. subtilis* and their putative functions.

**Table 1:** Putative Ars resistance proteins and homologous genes in *B. subtilis.*

As Resistance Proteins	Possible Genes with Homology in *B. subtilis*	Similarity (%)	Description	Gene Function Evidence	References
ArsH	*yhdA*	25.41%	NADPH-dependent FMN oxidoreductase	Experimental	[21,115,116,123]
ArsM	*ydaC*	28.70%	similar to N-methyltransferase	Putative	[21,90,117]
ArsN	-	-			[114]
ArsJ	-	-			[103,118]
ArsP	*ycgR*	23.55%	membrane protein similar to permease	Putative	[90,119]
ArsO	*czcO*	27.48%	flavin-containing monooxygenase, facilitates cation export via CzcD	Experimental	[103,121,124]
ArsT	*yumC*	26.88%	ferredoxin/flavodoxin-NAD(P) oxidoreductase	Experimental	[122,125]
ArsK	-	-			[103,120]

### 3.4. Overcoming Genetic Damage: DNA Repair and Stress Responses

It has been widely reported that As can cause DNA damage through distinct mechanisms, including oxidative damage, single- and double-strand breaks, and interference with repair systems. The type of DNA damage is dependent on the As species [68,105].

As has been reported to inhibit DNA repair in *E. coli* by affecting one or more steps in the post-replication repair pathway. This is based on the observation of decreased survival of WT or nucleotide excision repair-deficient *E. coli* cells exposed to UV light and subsequently to arsenite, whereas the addition of arsenite after UV irradiation of a *recA*-deficient strain did not show any further decrease in survival [126].

DNA repair is an important factor contributing to arsenic resistance in both eucaryotes and prokaryotes. Upregulation of DNA repair-related gene transcripts has been observed in arsenic-resistant bacteria such as *Thiomonas* sp. and *Herminiimonas arsenicoxydans* and in microbial communities from arsenic-contaminated environments [105].

**Figure 7:**
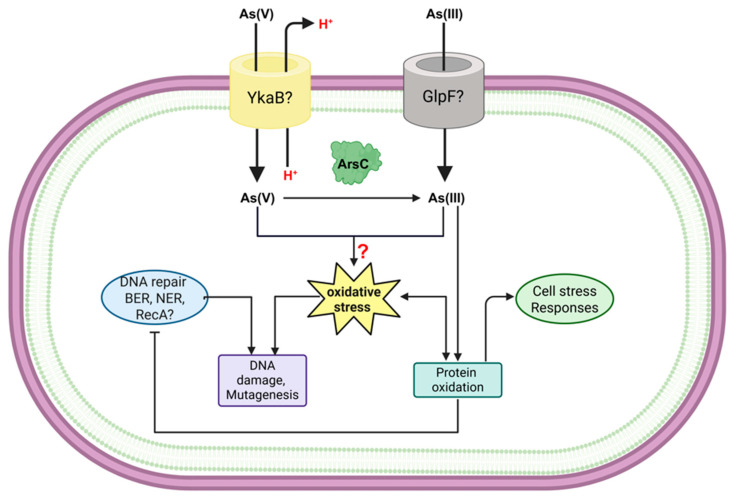
Hypothetical mechanism of ROS-promoted DNA damage, protein oxidation, DNA repair, and cell stress responses in *Bacillus subtilis*. Arsenate and arsenite are putatively internalized through YkaB and GlpF, respectively. Inside cells, arsenate can be enzymatically reduced to arsenite by ArsC. Following yet-to-be-discovered mechanisms, Ars(V) and Ars(III) elicit the production of oxidative stress. Oxygen radicals can impact the bacterial chromosome, inducing oxidative DNA damage and mutagenesis, which can be counteracted by DNA repair and recombination. Reactive oxygen species (ROS) and As(III) can damage proteins, interfering with DNA damage and inducing cell stress responses. BER: Base excision repair; NER: nucleotide excision repair. Created in BioRender. Valenzuela, L. (2025) https://BioRender.com/pbay3db. Image published on 23 September 2025.

In *B. subtilis*, DNA repair mechanisms and the mutagenic effects of some agents, including metals like chromium, have been studied [127,128]. However, studies on the *Bacillus* response to arsenic exposure have primarily focused on resistance mechanisms such as efflux, reduction, and oxidation, rather than the involvement of DNA repair enzymes. Only one study has highlighted the importance of superoxide dismutase in counteracting oxidative stress caused by As [129]. This lack of information presents an opportunity for future research into DNA repair systems in *Bacillus* species (Figure 7).

## 4. *Bacillus* Species Resistant to Arsenic: From Environmental Strains to Biotechnological Tools

### 4.1. Environmental Isolates of Arsenic-Resistant Bacillus

The prevalence of arsenic-resistant *Bacillus* species is notable in various environments and geographic regions, both contaminated and uncontaminated, suggesting that this bacterial genus possesses well-conserved resistance mechanisms and significant adaptive strategies. A wide variety of *Bacillus* species have been identified in environmental isolates from different sites, including aquifers, sediments, soil, hot springs, industrial effluents, mines, desert ecosystems, mining tailings, hyperalkaline lakes, the rhizosphere, and in association with plants (Table 2). While several reviews have focused on these isolates and their potential for bioremediation [31,111,130,131], this section focuses on the resistance mechanisms described in these isolates without ignoring the biotechnological potential of the particular species to which these isolates belong.

The mechanisms responsible for As resistance in the isolates listed in Table 2 include detoxification through efflux pumps, reduction, oxidation, and immobilization, all of which are encoded by *ars*, *ase*, *aiox,* and *arr* operon genes found either on plasmids or on the bacterial chromosome [132]. Reduction of arsenate to arsenite by the *arsC* gene has been associated with resistance mechanisms in some isolates, such as *B. cereus* AG27, capable of withstanding arsenate at 40 mM and arsenite at 35 mM. This high resistance was attributed to the presence of a 5.14 Kbp plasmid containing the *arsC* gene, which encodes an arsenate reductase highly similar to that of *B. subtilis* [133].

In *Bacillus firmus* L-148, induction of the *arsC* gene was also observed in the presence of 10 mM As(III), although arsenic in this isolate was shown to induce the expression of resistance genes at different levels: *arsA* > *arsB* > *aioS* > *arsR* > *arsD* > *arsC*. Additional determinants, including *ars mem* (arsenical membrane protein), *arsC rel* (As(V) reductase-related protein), *acr-3-2* (As(III) efflux pump), and *ars trans* (arsenic transporter), were detected in this study [57]. Further induced factors in this isolate included stress response and stage IV sporulation proteins, as well as superoxide dismutase [57].

Reduction of arsenate to arsenite, by arsenate reductases, appears to be a common resistance mechanism among several isolates, as a key step preceding the expulsion of As(III) by efflux pumps. This mechanism has been described in *B. licheniformis* DAS-2, *B. firmus* L-148, *B. simplex*, and *B. megaterium*, where the presence of the *arsB* gene was identified [41,42,57,134,135]. Gu et al. 2018 found that some *Bacillus* sp. isolates carrying the gene for the Acr3 efflux pump exhibited higher arsenic tolerance (minimum inhibitory concentration, MIC, of 42 mM NaAsO_2_) than *B. megaterium* isolates carrying the *arsB* efflux pump gene (MIC of 24 mM NaAsO_2_) [136].

In some isolates, like *Bacillus* sp. IIIJ3-1, in addition to As(III) efflux systems (*arsB* and *acr3*), the presence of the *arrA* gene (coding for a respiratory As(V) reductase) and the *aioB* gene (coding for an As(III) oxidase) was also detected. This isolate demonstrated arsenic sequestration and cytosolic arsenate reduction capabilities, although no *arsC* gene was detected, suggesting either an alternative As(V) reduction mechanism or sequence diversification of the *arsC* gene.

In some *Bacillus* species, such as *B. paralicheniformis* ZAP17 and *B. altitudinis* ZAP62, the *arsA* and *arsD* genes have also been identified as part of the *arsRBCDA* operon. The *arsA* gene encodes an ATPase that powers the arsenic pump, while the *arsD* gene encodes a metallochaperone that binds As(III) and delivers it to the ArsA subunit of the efflux pump [111].

An alternative mechanism to counteract the toxic effects of As in some *Bacillus* isolates involves the production of siderophores or, in the case of As, “arsenophores” that are ligands capable of chelating arsenic salts [137]. None of the *Bacillus* isolates described in Table 2 were found to possess genes encoding arsenic methyltransferases. However, the *Bacillus* sp. MD1 isolate, recovered from a golf course, was found to carry the *arsI* gene, responsible for As demethylation. It is well-known that arsenical herbicides are commonly used on golf courses, so the presence of this enzyme in the isolate may be related to the ability to demethylate As, thereby detoxifying it into its inorganic form [91].

As noted above, the presence of As resistance genes in *Bacillus* isolates has been reported both on the genome and on plasmids. Bacteria like *Bacillus* sp. AG24, *Bacillus* sp. AGM13, *B. cereus* AG24 [135], and *B. cereus* AG27 [133] carry these determinants in plasmids, while in most arsenic-resistant *Bacillus* bacteria, the genes are chromosomally encoded. Table 2 lists different environmental strains of *Bacillus* and the locations where they were found.

**Table 2:** Identification and sources of *Bacillus* species isolated from arsenic-polluted and other environments.

Isolation Site Contaminated with Arsenic	Identified Species	References
**Aquifers contaminated with arsenic**		
West Bengal, India	*Bacillus indicus* sp. *nov.*	[138]
West Bengal, India	*Bacillus arsenicus* sp. *nov.*	[139]
Brahmaputra River basin, India	*Bacillus* sp. *IIIJ3-1*	[56]
Groundwater wells of Hazrapara and Ghoshpara locality of Beldanga, Murshidabad (Distt.), West Bengal, India	*Bacillus* sp.	[135]
Well water, Taif City, Kingdom of Saudi Arabia	*Bacillus fusiformis* strain EA2, *Bacillus cereus* strains EA4, EA5 y EA6	[140]
**Water bodies**		
Spring of water in Qorveh county, Kurdistan province, Iran	*Bacillus flexus A-12*	[59]
Geothermal systems of Araró, Mexico	*Bacillus altitudinis ZAP62*, *Bacillus paralicheniformis ZAP17*	[111]
Surface and groundwater samples, Rautahat District of Nepal	*Bacillus smithii*, *Bacillus cereus*	[141]
**Lake and lagoon sediments**		
Lake Oliveri–Tindari lake sediments, Italy	*Bacillus* sp.	[142]
Sediments of the Orbetello Lagoon, Italy	*Bacillus* sp. *ORAs2*	[143]
Sediments of Mono Lake, California	*Bacillus arsenicoselenatis*, sp. *nov.*, *Bacillus selenitireducens*, sp. *nov.*	[55]
Sediments of Mono Lake, California	*Bacillus beveridgei* sp. *nov.*	[144]
Aquifer sediments of Datong Basin	*Bacillus cereus* strain XZM002	[145]
**Soils**		
Soil from Alkaline Crater Lake, Lonar, Maharastra, India	*Bacillus firmus L-148*	[57]
Soil from Alps, Italy	*Bacillus* sp.	[146]
Agricultural soil and mining origin soil, Guanajuato, Mexico	*Bacillus simplex*, *Bacillus simplex strain LRV34*, *Bacillus muralis strain HlS3200905*, *Bacillus simplex strain Md1-25*, *Bacillus megaterium strain LB11*, *Bacillus* sp. *strain Whitaker B12*, *Bacillus megaterium strain 1S7*	[134]
Soil from Beijing, China	*Bacillus idriensis*	[147]
Soil of the Panki thermal power plant, Kanpur, Uttar Pradesh, India	*Bacillus cereus AG27*	[133,135]
Soil of Miyazaki Prefecture, Japan	*Bacillus megaterium* strain UM-123	[148]
Soil of Unnao district of Uttar Pradesh (India)	*B. megaterium* and *B. pumilus*	[149]
Soil of Uttar Pradesh, India	*B. subtilis*	[69]
Soil of Shanxi Province in Northwest China	*Bacillus thuringiensis* sp. *IAM*	[150]
Soils from cattle dip sites	*Bacillus* sp. *CDB3*	[151]
Golf course flooring, Florida, United States of America	*Bacillus* sp. *MD1*	[91]
**Industrial and domestic effluents**		
Industrial effluent treatment plant, Vapi, India	*Bacillus* sp. *DJ-1*	[152]
Industrial wastewater from the chemical industry in Sheikhupura, Pakistan	*Bacillus licheniformis*	[58]
Wastewater from outskirts of Lahore, Pakistan	*Bacillus subtilis* L24, *Bacillus safensis* L26 and *Bacillus subtilis* T23	[153]
Sediment from an effluent drain from a glass-manufacturing plant	*Bacillus selenatarsenatis* sp. *nov.*	[154]
Tannery effluents of Savar, Bangladesh	*Bacillus anthracis*	[155]
**Mines**		
Sediment from mining site, Hokkaido, Japan	*Bacillus cereus*, *Bacillus pumilus*,	[156]
Ore sample, Bundugurang opencast uranium mine, India	*Bacillus altitudinis 41KF2a*	[157]
Acid mine drainage site in Sabah, Malaysia	*Bacillus thuringiensis*	[158]
Soil samples from a gold mining area, Paracatu, Minas Gerais, Brazil	*Bacillus cereus* (*P2Ic*, *P1C1Ib*)	[132]
**Desertic ecosystems**		
Mongolian desert soil	*Bacillus safensis MS11*	[159]
**Rizosphere**		
Rhizosphere of the plant *Prosopis laevigata* from Villa de la Paz, located in the mining district of Santa María, in the State of San Luis Potosi, Mexico	*Bacillus megaterium* Jz11, *Bacillus aryabhattai B8W22*, *Bacillus simplex 98AIA*, *Bacillus axarquiensis, Bacillus malacitensis CECT*, *Bacillus subtilis CYBS15*, *Bacillus vallismortis DSM 11*,*031*, *Bacillus endophyticus 70BC7*, *Bacillus niacini IFO15566*,	[137]
Rhizosphere of *Amaranthus viridis*, Bihar Sharif, India	*Bacillus licheniformis DAS-2*	[41]
Rhizosphere of *Pteris vittata L*. in Hanyuan, Sichuan, China	*Bacillus indicus*, *Bacillus cereus*, *Bacillus muralis*, *Bacillus subtilis*, *Bacillus megaterium*, *Bacillus* sp.	[136]
Rhizospheric soil samples from the Baruipur district, West Bengal, India	*Bacillus flexus NM02*	[160]
**Other sites**		
Surface of used polyethylene terephthalate (PET) bottle fragments	*Bacillus* sp. *EIKU23*	[161]
Sludge of a sewage treatment plant, Taiwan, China	*Bacillus cereus* OSBH5	[162]
Mangrove sediment, Matang Mangrove Forest, Perak, Malaysia	*Bacillus* sp. CCB-MMP212	[163]

### 4.2. Harnessing Bacillus for Bioremediation and Sustainable Agriculture

The mechanisms described in this review unveil the strong potential that the microorganism of the *Bacillus* genus possesses for the development of bioremediation, phytoremediation, and environmental monitoring strategies in arsenic-contaminated sites.

One of the advantages of this genus lies in its ability to generate spores, which are differentiated cell forms with characteristics for enduring adverse environments that are inhospitable to other organisms. Additionally, *Bacillus* has been documented to promote plant growth through the production of volatile compounds; meanwhile, associations such as the Food and Drug Administration (FDA) have classified bacteria such as *B.sutilis*, *B. licheniformis*, *B.clausii*, and *B. coagulans* as safe given that they do not produce toxins or are pathogenic, and can therefore be used as probiotics or for enzyme production [164,165,166].

The main uses of *Bacillus* strains in biotechnology also include immobilizing enzymes on spore surfaces, improving stability and reuse, producing biofuels such as butanediol and ethanol, biological compounds such as vitamins and antibiotics, and even developing vaccines that use *B. subtilis* as an antigen presenter on the cell or spore surface. All of this highlights the importance of understanding the ability of *Bacillus* strains that may encounter the metalloid in both industrial and agricultural water to detoxify arsenic and their role in mitigating toxicity in agricultural food production. Some studies have addressed the possibility of using *Bacillus* strains for As removal in water or soil, and others have addressed their contribution to reducing toxicity to the microenvironment through various interactions with microorganisms or plants.

The biosorption, immobilization, and capture capacities of various *Bacillus* species, whether through living cells, inactive biomass, or modified biomass, can be applied to immobilize As in soils, remove it from aqueous solutions and effluents, and mitigate toxicity in ecosystems, thereby contributing to the proliferation of other species. A clear example is the non-living biomass of *B. cereus* Sz2, which has a high As(III) biosorption capacity of 153.41 mg/g and can be reused at a rate of up to 94% after desorption at an acidic pH of 1. This makes it ideal for arsenic removal in mine effluents [36]. Similarly, the *B. salmalaya* 139SI strain can be used as a biosorbent for As(V) in aqueous solutions, as it reaches a maximum removal efficiency of 92% and a recovery rate of 93% after ten desorption cycles [46]. *B. thuringiensis* WS3 biomass has also been used to remove As(III) from wastewater via passive adsorption of metal ions through functional groups in the cell wall, such as amino, phosphate, hydroxyl, and carboxylate [38]. A mixture of bacteria, including *B. amyloliquefaciens*, *B. cereus*, *B. velezensis*, and *Bacillus* sp., which was immobilized with loaded biocarbon, demonstrated the ability to immobilize arsenic, lead, and cadmium in soils through complexation, ion exchange, oxidation, and precipitation mechanisms [167].

The ability of certain *Bacillus* species to control the mobility of arsenic in soil is applied in environmental cleanup efforts [168]. Moreover, plant growth-promoting rhizobacteria (PGPR) not only lessen metal toxicity in plants but also prevent arsenic from reaching the consumable portions of crops, which is crucial for ensuring food safety [168].

Some strains of this genus, isolated from arsenic-contaminated agricultural soils, including *Bacillus* sp. ZH16, helped reduce arsenic translocation and promoted wheat plant growth when combined with biogenic molybdenum nanoparticles (MoNPs) while triggering the production of indole-3-acetic acid (IAA), phosphate solubilization, and ACC deaminase activity [169]. In an arsenic-contaminated agricultural field in Durgapur, India, a native *B. cereus* (PMM6) isolate exhibited resistance to arsenate (75 mM) and arsenite (25 mM), presumably through mechanisms of biosorption and bioaccumulation of these As forms [37]. This strain also displayed plant growth-promoting traits such as indole-3-acetic acid production, ACC deaminase activity, phosphate solubilization, and siderophore production, facilitating rice growth under arsenic stress [37,169], reducing arsenic mobilization, and thus preventing the generation of ROS.

It has also been reported that inoculation with *B. subtilis* S4 combined with iron nanoparticles helped mitigate arsenic-induced toxicity in *Cucurbita moschata* seeds by enhancing peroxidase (POD) and superoxide dismutase (SOD) activity [170], reducing levels of hydrogen peroxide, malonaldehyde, and electrolyte leakage. In other report, inoculation with *B. amyloliquefaciens* (NBRISN13) in combination with feldspar was found to reduce arsenite translocation and decrease As content in rice grains (52–72%). This treatment alleviated oxidative stress via modulation of enzymatic activity and phytohormone production, enhancing plant growth and yield [152,171]. *B. amyloliquefaciens* SN13 also reduced arsenic toxicity in rice by modulating the expression of carbohydrate metabolism and arsenic stress-related genes [45].

A consortium formed by *B. licheniformis* (NDSA24R), *Priestia endophytica* NDAS01F, and *Priestia flexa* NDAS28R isolated from Nadia, West Bengal, reduced As concentration in rice plants by lowering bioconcentration and translocation factors. This consortium induced systemic acquired resistance, stimulated sulfur metabolism and cell wall synthesis, and reduced oxidative stress in treated plants [43].

Another study evaluated the combination of *B. faecalis* and enriched composted biochar (ECB) to alleviate arsenic stress in maize plants. Results showed that this combination enhanced maize growth under arsenic exposure by regulating antioxidant production and reducing arsenic uptake by roots and leaves [172]. *B. pacificus* (AKS1), an arsenic-tolerant strain isolated from arsenic-contaminated groundwater in West Bengal, India, displays promising traits for environmental bioremediation. This species tolerates multiple metals and arsenic, growing in 20 mM arsenate and 10 mM arsenite. Genome analysis revealed the presence of As resistance genes, including *arsC*, *arsB*, and *arsR*. It also exhibited arsenic adsorption capacity and production of plant growth-promoting metabolites such as indole acetic acid (IAA), gibberellic acid (GA), and proline, along with nitrogen fixation capabilities [47].

### 4.3. Engineering Biosensors: Precision Detection of Arsenic

Some studies have employed genetic engineering to enhance the bioremediation potential of *B. subtilis* or to develop As biosensors. One notable work demonstrated the feasibility of heterologous expression in *B. subtilis* 168 of the arsenite-S-adenosylmethionine methyltransferase enzyme (CmarsM) from the thermophilic alga *Cyanidioschyzon merolae*, enabling the conversion of inorganic arsenic into dimethylarsinate and trimethylarsine. This modified strain was tested in arsenic-contaminated organic manure compost, resulting in significantly increased arsenic volatilization [173].

Understanding the *ars* operon has also been crucial in the development of As biosensors, leveraging the operon’s specific response to arsenic. Since the *ars* operon is regulated by the Ars repressor protein, genetic engineering allows the fusion of the promoter region (including the ArsR binding site) with a reporter gene. As(III) leads to repressor dissociation, allowing reporter expression. Biosensors in *B. subtilis* have been created by fusing the *ars* promoter to reporter genes such as β-galactosidase (*lacZ*) [70], luciferase, catecol-2,3-dioxygenase (*xylE*) [174,175], and GFP [71].

Furthermore, *B. subtilis* spores have been exploited to develop spore-based biosensors for easy portability, storage, transport, and field use. These spores germinate in the presence of samples and produce vegetative cells capable of responding to arsenic within a relatively short time, outperforming biosensors based on other bacteria. For instance, the spore-based biosensor using plasmid pMutin3-*ars-lacZ* retained detection ability after at least six months of storage at room temperature and could detect As concentrations as low as 1 × 10^−7^ M in freshwater and serum samples [71,175,176]. These spores can germinate in the presence of the sample and generate vegetative cells capable of responding to As in a relatively short time compared to biosensors of other bacteria. The As detection system based on *B. subtilis* spores with the pMutin3-*ars-lacZ* plasmid stored at room temperature maintained their arsenic detection capability after at least 6 months [70], being able to sense As concentrations from 1 × 10^−7^ M in freshwater and serum samples [176]. The spores of the pAD123-*ars-gfpmut3a* biosensor were able to germinate and emit fluorescent signals in less than 4 h in the presence of water artificially contaminated with arsenic from a concentration of 100 µM As(III) [71]. For the biosensor with the *xylE* reporter gene, it was observed that the sensitivity of the spore-based biosensor was low at an arsenic concentration of 1 mM but was strong and visible to the naked eye at a concentration of 5 mM As [175]. Although improvements to this biosensor allowed for decreased response times and minimum detection concentrations of As of up to 0.54 µg/L after 2 h of the addition of the enzymatic substrate catechol.

**Figure 8:**
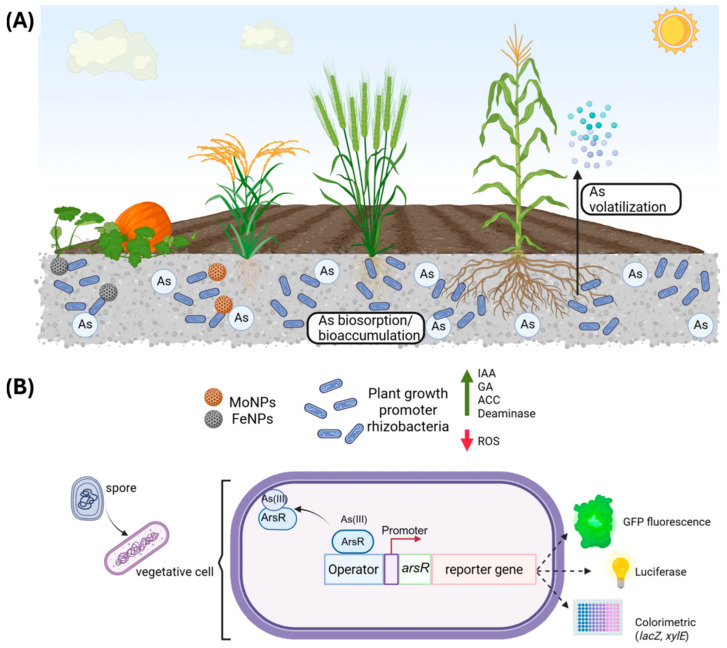
Biotechnological applications of *Bacillus* species in As remediation and detection. (**A**) As remediation mechanisms. *Bacillus* cells immobilize As via biosorption/bioaccumulation or mobilize it through volatilization. Plant growth promotion rhizobacteria, producing indol acetic acid (IAA), gibberellic acid (GA), 1-aminocyclopropane-1-carboxylate (ACC) deaminase activity, phosphate solubilization, and nanoparticle synergy (Mo/FeNPs), mitigate As toxicity in crops like rice, wheat, pumpkin, and maize. (**B**) Engineered As biosensors. Genetically modified *ars* operon enables As-responsive reporter systems (GFP, lacZ, xylE). Spore-based biosensors offer portable detection for environmental/clinical monitoring. Created in BioRender. Valenzuela, L. (2025) https://BioRender.com/lzcilrb. Image published on 23 September 2025.

The use of biosensor spores has allowed the incorporation into a portable centrifugal microfluidic platform system in which quantitative response was achieved in an incubation time of 2.5 to 3 h, using small sample volumes and reagents [177]. Figure 8 summarizes several key biotechnological applications addressed in this section.

## 5. Concluding Remarks

*B. subtilis* and related species possess a multiplicity of strategies to detect and counteract the noxious effects of arsenic. While these bacteria rely on strategies of biosorption and transformation that prevent this metalloid from penetrating cells, they can deploy specific mechanisms to cope with its damaging effects [68,72,73]. Accordingly, evolution has equipped these species with genetic determinants that are specifically induced by As to generate sophisticated efflux pumps and reductases. This review has summarized the current knowledge of the complex, multi-faceted defense mechanisms these bacteria use. These strategies include immobilization and redox transformation, as well as highly regulated intracellular detoxification via the *ars* and *ase* operons. The core finding is that *Bacillus* does not deploy a single mechanism, but rather a coordinated arsenal. The initial line of defense involves biosorption and potential extracellular enzymatic transformation, which limits cellular uptake [31]. Once internalized, the *ars* operon—with its phage-derived, co-regulated components (*arsR*, *yqcK*, *arsB/Acr3*, and *arsC*)—and the chromosomally encoded *ase* operon (*aseR* and *aseA*) work together to reduce, sequester, and actively efflux the toxic metalloid. Our comparative genomic analysis highlights the diversity in genetic arrangements of these operons across different *Bacillus* species, underscoring an evolutionary trajectory shaped by vertical inheritance and horizontal gene transfer, particularly from mobile genetic elements like the SKIN prophage [74].

It is important to note that, despite the significant focus on expulsion and enzymatic detoxification, arsenic’s insidious effects extend to genetic material itself. Exposure to arsenic induces oxidative stress and direct DNA damage, posing a fundamental threat to genomic integrity. While the specific DNA repair pathways activated by *Bacillus* in response to arsenic-induced lesions remain largely unknown, the bacterium’s survival in highly contaminated environments implies their existence. Future research must prioritize elucidating these repair mechanisms, as enhancing genomic stability could be key to engineering hyper-resistant strains for bioremediation.

Environmental isolates of *Bacillus* further demonstrate the adaptability and biotechnological potential of these systems, showcasing diverse resistance strategies across contaminated ecosystems [111,131]. The in-depth understanding of resistance mechanisms employed by *Bacillus* has made it possible to exploit its biotechnological potential in bioremediation and sustainable agriculture [37,168,169,170,171]. Their ability to sporulate, their safety profile in numerous species, and their metabolic versatility make them ideal for practical applications. They are used as efficient biosorbents to remove As from wastewater, as inoculants to immobilize As in soil, and importantly, as plant growth-promoting bacteria (PGPR) to mitigate As toxicity in crops such as rice and wheat. Several *Bacillus* strains reduce the translocation of the metalloid to the edible parts of plants, thereby ensuring food safety, and improve plant growth by producing phytohormones, solubilizing phosphate, and exhibiting ACC deaminase activity. Therefore, arsenic-resistant *Bacillus* species are a powerful and versatile tool for developing bioremediation strategies and ensuring agricultural productivity in contaminated soils. Beyond bioremediation, *B. subtilis* emerges as an ideal platform for the development of next-generation As biosensors [70,71,176,177]. Its ability to form ultra-stable spores allows for the creation of portable, long-lasting, field-ready devices capable of detecting trace concentrations of As(III) in water and soil. Genetic engineering of the *ars* operon, fused to reporters such as GFP or luciferase, makes these bacteria sensitive, economical, and ecologically sustainable tools for real-time environmental and clinical monitoring.

Key challenges remain, such as elucidating the full diversity of As uptake pathways in bacteria from the genus *Bacillus* and understanding the ecological implications of horizontal gene transfer in As resistance dissemination. Future research should explore the integration of these microbial systems into bioremediation technologies, leveraging genetic engineering to enhance As sequestration, volatilization, or biosensor development. Additionally, the role of *Bacillus* in plant-associated As mitigation warrants deeper investigation to optimize sustainable agricultural practices in contaminated regions.

Addressing the global challenge of arsenic contamination requires a concerted and interdisciplinary strategy. Translating the knowledge of *Bacillus* molecular biology into practical applications necessitates synergistic collaboration among microbiologists, genetic engineers, environmental scientists, materials science experts, agronomists, artificial intelligence developers, electronic engineers, and data scientists. The integration of genomics, synthetic biology, and advanced materials engineering is essential for designing next-generation bioremediation strategies and high-precision biosensors. Consequently, the soil bacterium *B. subtilis*, with its sophisticated and evolutionarily refined arsenic resistance machinery, represents a powerful and versatile platform for developing safer and more sustainable environmental solutions.

## Data Availability

No new data were created or analyzed in this study. Data sharing is not applicable to this article.

## Supplementary Materials

The following supporting information can be downloaded at: https://www.mdpi.com/article/10.3390/ijms262110277/s1 [178].
