# Supplementary material for: Mechanisms of Arsenic Interaction in Bacillus subtilis and Related Species with Biotechnological Potential
Source: Int J Mol Sci. 2025 Oct 22;26(21):10277. doi: 10.3390/ijms262110277 (PMC12607644; doi:10.3390/ijms262110277)
Supplement: Supplementary file 1 [file ijms-26-10277-s001.zip › ijms-3832702-supplementary.pdf]

Supplementary Material.

Review

Mechanisms of arsenic interaction in *Bacillus subtilis* and related species with biotechnological potential.

Luz I. Valenzuela-García[1], María Teresa Alarcón-Herrera[1*], Elizabeth Cisneros Lozano[2], Mario Pedraza-Reyes[3] and Víctor M. Ayala-García[2*].

[1] Department of Sustainable Engineering, Advanced Materials Research Center (CIMAV), Durango, Mexico. P.C. 34147; luz.valenzuela@cimav.edu.mx
[2] Faculty of Chemical Sciences, Juárez University of Durango State, Durango, Mexico. P.C. 34120; 66554@alumnos.ujed.mx
[3] Department of Biology, University of Guanajuato, Guanajuato, Mexico; pedrama@ugto.mx
* Correspondence: teresa.alarcon@cimav.edu.mx (M.T.A.-H.); victor.ayala@ujed.mx (V.M.A.-G.)

Table S1. Access IDs for nucleotide and amino acid sequences of As resistance components reviewed in this study. All entries are available on the NCBI portal.

Reference genome	Gene name	Function	NCBI Nucleotidic sequence ID	NCBI Amino acid sequence ID
Bacillus subtilis 168	*arsR*	Transcriptional repressor	BSU_25810	NP_390458.1
	aseR	Transcriptional repressor	BSU_05330	NP_388414.1
	yqck	Putative C-As lyase	BSU_25800	NP_390457.2
	arsB	Arsenite efflux pump	BSU_25790	NP_390456.2
	aseA	Arsenite efflux pump	BSU_05340	NP_388415.1
	arsC	Arsenate reductase	BSU_25780	NP_390455.1
Bacillus licheniformis ATCC 14580	*arsR*	Transcriptional repressor	NZ_CP140161.1:2772679-2773026	WP_003182415.1
	arsB	Arsenite efflux pump	NZ_CP140161.1:2773150-2774445	WP_003182413.1
	arsC1	Arsenate reductase	NZ_CP140161.1:1540137-1540493	WP_003185068.1
	arsC2	Arsenate reductase	NZ_CP140161.1:2774468-2774887	WP_003182412.1
Bacillus anthracis Ames 0580	*arsR1*	Transcriptional repressor	NZ_CP140161.1:2772679-2773026	WP_000046014.1
	arsR2	Transcriptional repressor	NC_007530.2:c2945317-2945012	WP_000207867.1
	arsR3	Transcriptional repressor	NC_007322.2:c174516-174223	WP_000152670.1
	arsI/cadI	Putative C-As lyase	NC_007530.2:c2944952-2944515	WP_001271033.1
	arsB	Arsenite efflux pump	NC_007530.2:2943456-2944496	WP_000826566.1
	arsC	Arsenate reductase	NC_007530.2:2943026-2943430	WP_000428348.1
Bacillus cereus FORC047	*arsR*	Transcriptional repressor	CP017060.1:3052928-3053233	WP_002026021.1
	arsI/CadI	Putative C-As lyase	NC_004722.1:c3123626-3123189	WP_089171391.1
	arsB1	Arsenite efflux pump	NZ_CP017060.1:598313-600679	WP_000826566.1
	arsB2	Arsenite efflux pump	NZ_CP017060.1:3051371-3052411	WP_000438018.1

	arsC	Arsenate reductase	NZ_CP017060.1:3050941-305	WP_089171389.1
Bacillus thuringiensis ATCC 10792	*arsR1*	Transcriptional repressor	NZ_CM000753.1:2887969-2888274	WP_002026021.1
	arsR2	Transcriptional repressor	NZ_CM000753.1:2888335-2888772	WP_000918378.1
	arsI/cadI	Putative C-As lyase	NZ_CM000753.1:2888335-2888772	WP_001271003.1
	arsB	Arsenite efflux pump	NZ_CM000753.1:c491484-489118	WP_000796571.1
	arsC	Arsenate reductase	NZ_CM000753.1:2889852-2890256	WP_001284548.1
Bacillus amyloliquefaciens GKT04	*arsR1*	Transcriptional repressor	NZ_CP072120.1:c777890-776778	WP_052125186.1
	arsR2	Transcriptional repressor	NZ_CP072120.1:1587401-1587751	WP_025285481.1
	arsR3	Transcriptional repressor	NZ_CP072120.1:c3857735-3857433	WP_003153841.1
	arsB1	Arsenite efflux pump	NZ_CP072120.1:1342619-1343950	WP_012118610.1
	arsB2	Arsenite efflux pump	NZ_CP072120.1:1587764-1589062	WP_061581630.1
	arsB3	Arsenite efflux pump	NZ_CP072120.1:2356009-2356971	WP_039062684.1
	arsC	Arsenate reductase	NZ_CP072120.1:c1000525-1000169	WP_003151843.1
Escherichia coli K-12	*arsR*	Transcriptional repressor	NC_000913.3:3648528-3648881	NP_417958.1
	arsB	Arsenite efflux pump	NC_000913.3:3648935-3650224	NP_417959.4
	arsC	Arsenate reductase	NC_000913.3:3650237-3650662	NP_417960.1
Acidithiobacillus ferrooxidans ATCC 23270	*arsR*	Transcriptional repressor	CP001219.1: 2543945-2544301	WP_012537435.1
Corynebacterium glutamicum ATCC 13032	*arsR*	Transcriptional repressor	NC_006958.1:1594430-1594789	WP_011265756.1
Bacillus sp. MD1	*arsI*	C-As lyase	KF899847.1:1301-1786	AIA09488.1
Thermomonospora curvata DSM 43183	*arsI*	C-As lyase	CP001738.1:4741190-4741645	ACY99683.1
Saccharomyces cerevisiae	*acr3p*	Arsenite efflux pump	NM_001184298.1	NP_015527.1
Staphylococcus aureus	*arsC*	Arsenate reductase	NC_007795.1:809502-809858	WP_154293969.1
Nostoc sp. PCC 7120	*arsI*	Putative C-As lyase	BA000019.2:1293372-1293824	BAB73061.1

Construction of phylogenetic trees.

Multiple sequence alignment was performed using the MUSCLE algorithm implemented in MEGA version 12 [178], with default parameters. The software then identified the evolutionary substitution model that best fitted the data, which was subsequently applied for the construction of phylogenetic trees using the Maximum Likelihood (ML) method. To evaluate the robustness of the tree branches, a bootstrap analysis with 1000 replicates was performed [178].
